# A Step-by-Step Design Strategy to Realize High-Performance Lithium–Sulfur Batteries

**DOI:** 10.1021/acsaem.4c02457

**Published:** 2025-01-28

**Authors:** Matthew J. Dent, Sean Grabe, Steven J. Hinder, Mateus G. Masteghin, James D. Whiting, John F. Watts, Constantina Lekakou

**Affiliations:** Centre for Engineering Materials, Faculty of Engineering and Physical Sciences, University of Surrey, Guildford GU2 7XH, U.K.

**Keywords:** lithium−sulfur batteries, interlayer, silk fibroin, catholyte, experiments, multiscale simulations, multipore continuum digital twin simulations

## Abstract

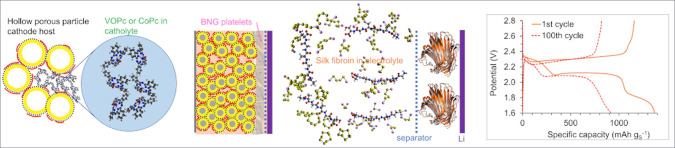

In order to increase the energy density and improve the cyclability of lithium–sulfur (Li–S) batteries, a combined strategy is devised and evaluated for high-performance Li–S batteries. It consists of the following steps to reduce the loss of active sulfur and sulfides migrating in the liquid electrolyte to the anode and add electrocatalyst groups in the cathode or catholyte: (i) A hollow porous nanoparticle coating cathode host with a pseudocapacitive PEDOT:PSS binder that also contributes to trapping polysulfides. (ii) A thin interlayer of B–N-graphene (BNG) nanoplatelets on the above cathode trapping polysulfides while participating in the electron transfer and acting as an electrocatalyst, thus ensuring that the trapped sulfides remain active in the cathode. (iii) Added semiconductor phthalocyanine VOPc or CoPc to form an electrocatalyst network in the catholyte, trapping polysulfides and promoting their redox reactions with Li^+^ ions. (iv) Added silk fibroin in the liquid electrolyte, which also suppresses dendritic growth on the lithium anode. This strategy is evaluated step-by-step in Li–S battery cells characterized experimentally and in simulations based on a multipore continuum physicochemical model with adsorption energy data supplied from molecular dynamics simulations. The thin BNG interlayer sprayed on the cathode proved a decisive factor in improving cell performance in all cases. A Li–S cell combining features from (i), (ii), and (iv) and with 45 wt % S in the cathode yields 1372 mAh g_S_^–1^ at first discharge and 920 mAh g_S_^–1^ at the 100th discharge after a cycling schedule at different C-rates. A Li–S cell combining features from (i), (ii), and (iii) and with 55 wt % S in the cathode yields 805 and 586 mAh g_S_^–1^ at the first and the 100th discharge, respectively.

## 1 Introduction

Current research in energy storage devices focuses on improving key performance indicators (KPIs) closely related to certain application fields, with electric vehicles (EVs) being the top application priority. These KPIs are (i) the energy density, aiming to increase the driving range of EVs and the operating range of portable devices; (ii) the power density, seeking to fast-charge EVs and other application devices. Supercapacitors exhibit high power density but still have low energy density, despite many efforts to tailor the pore size distribution of electrodes^1,2^ and functionalize carbon electrodes with pseudocapacitive groups.^3^ Hybrid battery-supercapacitor devices exhibit relatively good energy density, but their high-power supercapacitor part comprises only 10–15% of the total hybrid device energy.^3,4^ Alternatively, efforts to increase the power density of Li-ion batteries have been reported by developing carbonate electrolytes compatible with a hard carbon anode, aiming at raising the diffusion rate of Li^+^ ions in the anode^5^ to match the good Li^+^ ion diffusion coefficient in the latest high-performance NMC cathodes.^6^ Li-ion batteries are the main energy storage devices used for EVs, due to their high energy density, with the latest NMC batteries reaching 200–250 Wh kg^–1^.^7^ However, Li-ion batteries are reaching their upper limit with regard to energy density, and research is turning to alternative battery technologies.^7^ Attempts to replace lithium ions with cations based on abundant materials, such as ammonium ion^8,9^ and zinc ion^10^ have not yielded any higher energy densities so far. Hence, research for next-generation batteries has been directed to other cathode materials of high theoretical specific capacity, such as oxygen and sulfur. Metal-air batteries and fuel cells offer high theoretical energy density, for example, a theoretical energy density of 11140 Wh/kg_Li_ for Li–O_2_ batteries,^11^ but suffer from sluggish reaction kinetics, high overpotential, low efficiency, the need for expensive catalysts, the need for pure oxygen feed without moisture and CO_2_, and poor cyclability.^11,12^ Additional issues to be resolved concerning hydrogen fuel cells include green hydrogen production and the development of relevant safe infrastructure.^13^

The interest in lithium–sulfur (Li–S) batteries is due to their high theoretical energy density, over 2700 Wh kg_electrodes_^–1^, combined with the low cost and abundance of sulfur as the active cathode material. Considerations in translating the high specific capacity of sulfur to high cell energy density include maximizing the amount of sulfur in the cathode and minimizing the electrolyte-to-sulfur ratio, E/S.^14^ Combining an E/S = 6 μL mg^–1^ with a cell/sulfur mass ratio of 5.3, an energy density of 650 Wh kg_cell_^–1^ was achieved in pouch cells of Li–S battery with MoS_2_ cathode host.^15^ However, despite such high energy density, well above the performance of Li-ion batteries and supercapacitors^16−20^ that would have been ideal for long-range EVs, Li–S batteries still seem in the research stage with no manufacturers ready to adopt them for commercial applications.

Conductive cathode hosts have been devised to encapsulate the sulfur and accommodate its expansion when converted all the way to Li_2_S.^20^ A multipore continuum physicochemical model of the Li–S battery with liquid electrolyte 1 M LiTFSI in DOL/DME has been developed and employed by our group to aid in the design of the cathode host microstructure.^21−25^ First simulations using a digital twin based on this Li–S battery model identified several problems in Li–S batteries, many of which are closely associated with the cathode: (a) Sulfur in deep micropores remains undissolved and unutilized until the end of discharge, which frequently occurs in microporous hosts.^21−23^ At very low C-rates, a small amount of electrolyte solvent might soften the encapsulated solid sulfur or sulfides in such micropores, and redox reactions might occur in a semisolid state.^26−28^ (b) Sulfur and soluble sulfides in meso- and macropores can easily migrate to the anode during discharge and shuttle between cathode and anode during charge via the mechanisms of diffusion and drift current.^21^ This might occur even in high tortuosity graphene-based cathodes due to large pores between curled graphene platelets.^20,29,30^ (c) In many types of cathodes, sulfur and sulfide leaching takes place from different cathode locations toward the cathode surface facing the separator, resulting in exceeding the saturation concentration and precipitating, forming a thick layer there that remains mostly undissolved and generally does not participate in further redox reactions.^21,23,31^ (d) The presence of a pure lithium anode poses the risk of dendrite formation, as also in Li-ion batteries with a lithium metal anode.^4,32,33^

The following step-by-step strategy has been devised in the present study to address the above problems in Li–S batteries: (i) Use of hollow porous nanoparticle-based cathode host to retain sulfur in the nanoparticle core and thus reduce the “shuttling” effect. (ii) Develop a nanoplatelet interlayer between the cathode and separator to further reduce the “shuttling” effect and possibly eliminate it. (iii) Employ an effective electrocatalyst-type additive in the catholyte region to promote the Li–S redox reaction chain while also retaining sulfur and sulfides in the catholyte. (iv) Employ an additive in the electrolyte to suppress dendritic growth on the Li anode.

Hollow porous particle-based cathodes have been one of the first proposed solutions to reduce the “shuttling” effect in Li–S batteries^25,34^ with a reported specific capacity of 900 mAh g^–1^ after 100 cycles at 0.2 C^34^ and 700 mAh g^–1^ after 100 cycles at 1 C.^35^ Despite such high performance, there has been no follow-up in the commercialization of such sulfur cathodes, which might be attributed to two reasons: (a) The multistage synthesis method of the hollow porous particles based on a silica template, which needs to be removed typically with repeated washing steps using a highly toxic and corrosive HF aqueous solution.^34,35^ (b) The surprisingly high C-rate of reported cycling^35^ corresponds to a current density of at least 2 mA cm^–2^ at which dendritic growth is expected at the anode. According to Sand’s equation,^36^ the onset time for dendrite formation is estimated to be 225 s for a current density of 2 mA cm^–2^ in liquid electrolyte 1 M LiTFSI in DOL:DME 1:1 v/v, which is certainly within the discharge or charge time reported for the Li–S battery cycled at 1 C; this means that the reported batteries are at high risk of short-circuiting in any further charge–discharge cycling. (c) Furthermore, a very high E/S ratio, E/S = 61 to 122 μL mg^–1^, was required^35^ well above the recommended metrics for realizing a Li–S battery with good energy and power density with respect to cell mass.^14^ Other studies have used a commercially available material, Ketjenblack EC-600JD, with smaller hollow porous nanoparticles of about 30 nm as a cathode host in Li–S batteries that have achieved good specific capacity at first discharge in the range of 700–900 mAh g^–1^ at 0.1 C^37^ with a smaller amount of electrolyte at E/S = 6 to 11 μL mg^–1^.^23^ Hence, the Ketjenblack EC-600JD is the cathode host adopted in the present study. Furthermore, earlier research within our group replaced the typical PVDF binder in the cathode coating with PEDOT:PSS, which allows for more environmentally friendly cathode fabrication based on aqueous slurry, increases the specific capacitance by 7–9% due to the pseudocapacitance of PEDOT:PSS, and adsorbs the sulfur and polysulfides, significantly reducing the polysulfide migration to the anode, although there is still a small sulfur concentration at the anode.^38^

Much research in the past focused on modifications of the separator to suppress the “shuttling” effect.^39,40^ This includes functionalization or coating of the separator with chemical groups or materials that adsorb sulfur and polysulfides.^39−41^ Numerous papers have been published with DFT (density functional theory) and MD (molecular dynamics) simulations to determine the adsorption energy, *E*_ads_, of lithium sulfides and sulfur by different functional carbon or graphene substrates. These include *N*- and N–S- doping of graphene with *E*_ads_ in the range of 0.8 to 2.06 eV,^42^ Mo-functionalized graphene with *E*_ads_ around 2.3 eV,^43^ and B, N hexagonal codoped graphene with very high *E*_ads_ values of 4.4 eV, which is attributed to the strong Li···N and S···B interactions.^44^ An interlayer of 40 wt % functional boron-nitride nanosheets and 53 wt % graphene raised the specific capacity of a Li–S cell by only 10%.^45^ The disadvantage of that method is believed to be the low conductivity of the B–N nanosheets, which limited the electron transfer across the thickness of the interlayer and the utilization of any sulfur and sulfide trapped there. Hence, high conductivity B,N-codoped graphene (BNG) has been adopted in the present study, with three avenues of investigation: BNG-coated separator, BNG-coated cathode, or BNG coating on both the cathode and separator. The advantage of high conductivity BNG coating on the cathode is that any sulfur or sulfides adsorbed and trapped by the BNG layer will be in the path of electron transfer in the cathode and actively participate in the chain of redox reactions there. In particular, BNG has excellent potential for also acting as an electrocatalyst for these reactions.^46^

DFT simulations to predict the adsorption energy and electrocatalytic ability of different single atom catalysts (SACs) in terms of activation energy of the Li–S redox reactions in the presence of SACs of the type Me-N_4_- as part of a graphene structure yielded *E*_ads_ in the range of 3.3 to 4.5 eV for the best SACs V–N_4_-C and W–N_4_-C and *E*_ads_ in the range of 1.2 to 2 eV for Fe–N_4_-C and Co–N_4_-C with respect to the different lithium sulfides.^47^ Given that micropores of cathode hosts physically trap the sulfur and polysulfides, migration of these active substances to the anode takes place mostly via the meso- and macropores. Hence, the aim would be to functionalize the walls of such pores of porous cathode hosts with appropriate SACs to reduce the “shuttling” effect. However, DFT studies demonstrated that the *E*_ads_ values drastically decrease in pores greater than 1 nm, as one may generally consider that the adsorption energy falls with the reverse of distance away from the optimum position of the sulfide with respect to the functionalized substrate.^48^ Hence, a novel idea in the present study is to employ vanadyl phthalocyanine (VOPc) as an additive in the catholyte. VOPc comprises small molecules as semiconductor dipoles,^49^ which we envisage would form an SAC network in the catholyte within the meso- and macropores, bridging their walls, that would retain the sulfur and lithium sulfides locally in the pores and promote the redox reaction chain there, reducing leaching of active material toward the separator and anode. Apart from VOPc being an excellent electrocatalyst and Li_2_S_*x*_ absorbing means, a delicate balance is sought between the VOPc network offering sufficient conductivity to promote electron transfer but not too high conductivity to short circuit the cell, as the small VOPc molecules can easily be transported through the separator pores. Starting from the conductivity, σ, of multiwall carbon nanotubes (MWCNTs) that have been observed to cause short circuiting, σ_MWCNT_ = 100 to 2 × 10^5^ S m^–1^,^50,51^ a conductivity σ_VOPc_ = 0.5 to 1.5 × 10^–6^ S m^–1^ has been reported for VOPc,^52^ σ_CoPc_ = 2.4 × 10^–4^ S m^–1^ for CoPc,^53^ and even higher conductivity for CoPc tetramers σ_CoPc_ = 0.8 S m^–1^.^54^

With regard to the Li anode, silk fibroin was found to suppress dendritic growth in Li-ion batteries.^55^ MD simulations confirmed the role of the silk fibroin additive in the liquid electrolyte of Li–S batteries to passivate the Li anode by forming a coating monolayer linked via O···Li interactions with the Li surface and coordinated with a large number of lithium sulfide molecules with binding sites featuring O···Li and N···S interactions between the amino acid part of silk fibroin and the lithium and sulfide part of the Li_2_S_*x*_.^56^

**Figure 1:**
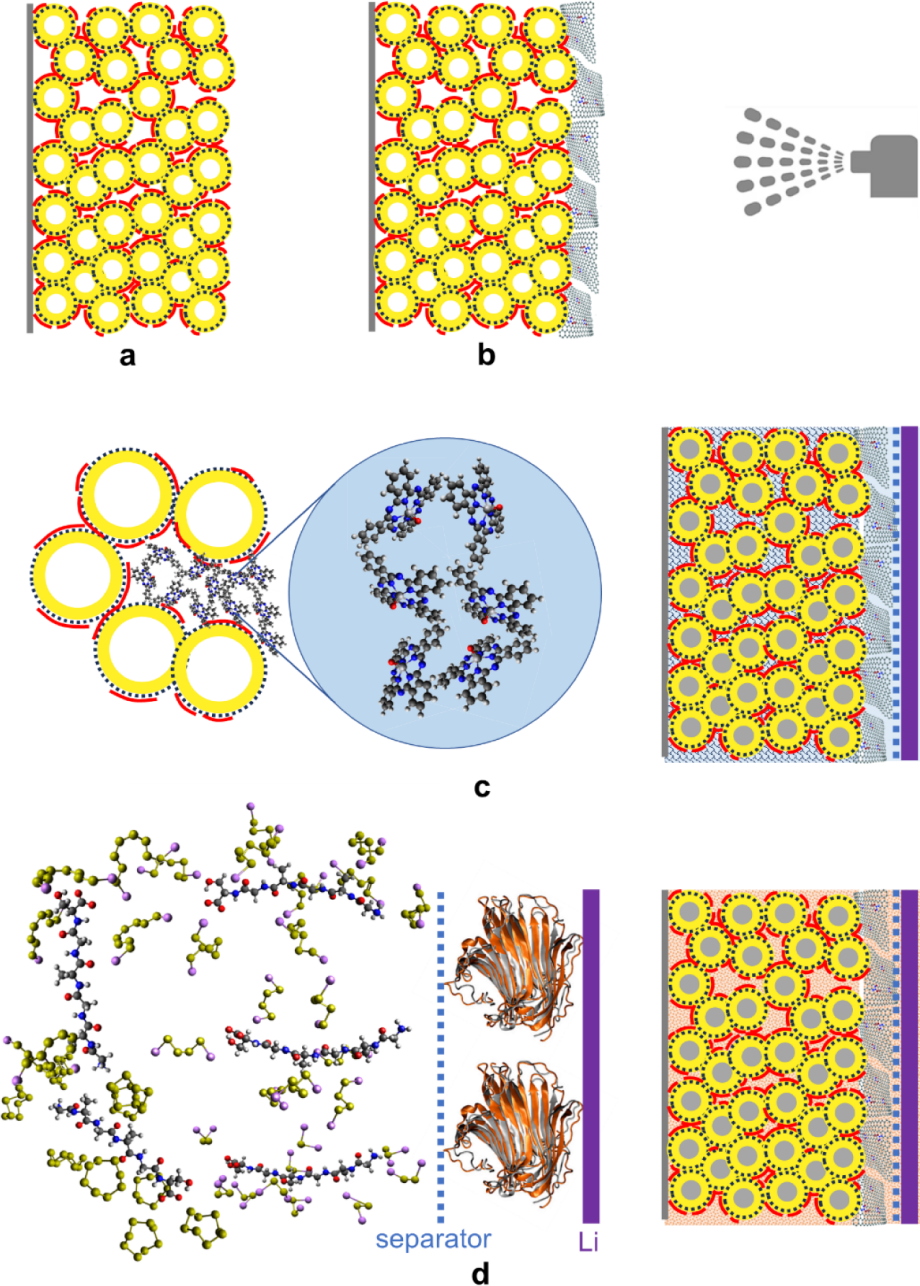
Diagram of the strategy steps to realize high-performance Li–S batteries in this study: (a) A hollow porous nanoparticle KB-based cathode with 10 wt % PEDOT:PSS binder part-coating the meso- and macropore walls (red in the diagram). (b) A KB-based cathode with a thin sprayed interlayer of BNG nanoplatelets on the cathode coating using SPE PEO as a binder for the interlayer. (c) A KB-based cathode with a thin BNG interlayer on the cathode surface and catholyte additive VOPc or CoPc nanodots considered to form an electrocatalyst network in meso- and macropores also attracting soluble sulfur and lithium sulfides. (d) A KB-based cathode with a thin BNG interlayer on the cathode surface and an electrolyte additive, silk fibroin, is considered to act in a dual role: forming a monolayer on the Li anode surface to suppress dendritic growth and coordinating with soluble sulfur and lithium sulfides.

Figure 1 outlines the strategy steps adopted in this study to realize high-performance Li–S batteries: (a) The cathode comprises a hollow porous nanoparticle coating based on Ketjenblack EC-600JD (henceforth referred to as KB) with 10 wt % pseudocapacitive PEDOT:PSS binder. (b) A KB-based cathode with PEDOT:PSS binder is still employed with a thin sprayed interlayer of BNG nanoplatelets on the cathode coating using, as interlayer binder, a solid polymer electrolyte (SPE); the aim is for the BNG interlayer to attract sulfur and sulfides and act as an electrocatalyst for the redox Li–S battery reactions. (c) Still employing the KB-based cathode with a thin BNG interlayer on the cathode surface, VOPc or CoPc nanodots are added to the catholyte with the aim for these semiconductor molecules to form an electrocatalyst network in the cathode meso- and macropores while also attracting soluble sulfur and lithium sulfides. (d) Still employing the KB-based cathode with a thin BNG interlayer on the cathode surface, silk fibroin is added to the liquid electrolyte with the aim to form a monolayer on the Li anode surface and suppress dendritic growth as well as coordinating with soluble sulfur and lithium sulfides. The experimental investigations are aided by simulations of discharge–charge of the Li–S cells with the corresponding component materials using the digital twin based on our continuum multipore physicochemical model. Such simulations predict the discharge–charge curves and also monitor the progress of the Li–S reactions and transport, dissolution, or precipitation of sulfur and polysulfides at different locations in the cell and pore sizes. Molecular modeling and simulations are deployed to provide any missing input data for the continuum simulations, such as adsorption energy data missing from the literature.

## 2 Methods

### 2.1 Experimental Methods

Ketjenblack EC-600JD (Lion Corporation, Japan), henceforth referred to as KB, was mixed with sulfur powder (Sigma-Aldrich, UK) and ground together for 30 min. The mixture was then dispersed in acetone for 30 min in an ultrasonic bath, followed by tip sonication for 10 min at room temperature. The dispersed sulfur-KB acetone slurry was magnetically stirred overnight until acetone evaporation. The resulting mix was further ground for 30 min and placed in an aluminum foil parcel, which was left in an oven for 3 h at 155 °C for sulfur infiltration in combined melt/vapor phase. The samples were weighed before and after the sulfur infiltration procedure to determine the sulfur mass retained in the KB host. The sulfur-infiltrated KB mix was ground for 30 min and added to an aqueous PEDOT:PSS solution (Clevios PH 1000, Heraeus, Germany), placed in an ultrasonic bath for 30 min, and left to stir on a magnetic stirrer to mix well and thicken. The viscous slurry was coated on a carbon-coated aluminum foil current collector (MTI, US) via the doctor blade technique at a blade gap of 250 μm. Cathode coatings of 45 and 55 wt % S in KB with 10 wt % PEDOT:PSS were manufactured in this manner. Alternatively, for higher sulfur contents, starting with the cathode coating of 45 wt % S melt/vapor infiltrated in KB, the additional sulfur was added via the solution method in the form of Li_2_S_8_ solution in DOL/DME 1:1 v/v. In this manner, cathodes of 55, 60, 65, and 70 wt % S were prepared with KB host and 10 wt % PEDOT:PSS. Li–S cells were fabricated using a two-part 316 SS case^4,17^ consisting of the above cathode disc of 19.2 mm diameter, Li anode disc of 19.2 mm diameter and 0.75 mm thickness (Sigma-Aldrich, UK), Celgard 2400 separator disc of 25 mm diameter, and electrolyte 1 M LiTFSI (lithium bis(trifluoromethanesulfonyl)imide) in DOL/DME (1,3-dioxolane/dimethoxyethane) 1:1 v/v with 0.8 M LiNO_3_ at E/S = 11 μL mg^–1^.

For the fabrication of the BNG interlayer, B,N codoped graphene (BNG) (Graphitene, UK) (according to manufacturer specifications: BNG powder of flakes of 1–5 atomic layers, with a lateral size of 0.5–5 μm, BET specific surface area SSA_BET_ > 500 m^2^ g^–1^ and composition: 80–90 at% C, < 5 at% O, 2.0–4.0 at% B, and 2.0–4.0 at. % N) was fully dispersed in acetonitrile with dissolved poly(ethylene oxide) (PEO) (Sigma-Aldrich, UK) of MW = 100 × 10^3^, and sprayed on the cathode surface as a very thin coating (with an undetected weight difference before and after the sprayed coating) of 90 wt % BNG and 10 wt % PEO binder. The Li–S cells with an S-KB cathode and BNG interlayer were filled with the liquid electrolyte, where three E/S ratios were tried in this study: E/S = 16, 11, and 8 μL mg^–1^.

Concerning the catholyte additives, cobalt phthalocyanine (CoPc) (Sigma-Aldrich, UK) or vanadyl phthalocyanine (VOPc) (Sigma-Aldrich, UK), 1 wt % of CoPc or VOPc of the cathode composition was added via the method of a 1:1 v/v solution of CoPc or VOPc in DOL/DME in the cathode, so it could permeate all micro-, meso-, and macropores in the cathode not filled with sulfur. Then the DOL/DME solution was left to evaporate, leaving the CoPc or VOPc in the cathode pores. Finally, the cell was filled with the specified amount of electrolyte in the specified E/S ratio.

The cells were subjected to electrochemical testing in the form of galvanostatic discharge–charge (GDC) cycles at different C-rates with respect to the sulfur content in the cathode. The sulfur-infiltrated KB powder was characterized by using a ThermoFisher Scientific Talos F200i scanning transmission electron microscope (STEM). Energy dispersive X-ray spectroscopy (EDX) maps were acquired by using a Bruker X-Flash detector. The standard KB-based cathode coating was subjected to SEM/EDX employing the instrument HR-SEM JEOL-7100 F. Cathodes and anodes were also characterized via SEM and SEM/EDX postmortem after being cycled according to the full cycle schedule applied in this study. The anodes were also characterized postmortem via X-ray photoemission spectroscopy (XPS) using a Thermo Scientific K-Alpha+ XPS instrument. XPS spectra were obtained in constant analyzer energy mode with pass energies of 200 and 50 eV and step sizes of 0.4 and 0.1 eV for survey and high-resolution spectra, respectively. A monochromated Al Kα X-ray source was used with a 400-μm radius spot. The obtained XPS spectra were analyzed using Thermo Scientific Avantage V5.980 software (ThermoFisher Scientific Ltd., Horsham, UK).

### 2.2 Numerical Methods

The digital twin based on the multipore continuum model, which was developed within our group for Li–S batteries^21^ and validated for different types of cathode microstructures and electrolyte 1 M LiTFSI in DOL/DME,^21−23^ was employed for simulations of the first discharge–charge cycle of three selected cells, which had a cathode of 45.4 wt % S-KB-10 wt % PEDOT:PSS. Two of these cells also had a BNG interlayer on the cathode. Finally, one of these two cells also had 0.8 wt % SF in the electrolyte. Input data for the dissolution/precipitation kinetics and the saturation concentrations of sulfur and lithium sulfides were extracted from previous experimental studies.^57,58^ A pseudocapacitance model was included for the PEDOT:PSS binder as presented in a recent study.^38^ The adsorption energies of sulfur and sulfides by the substrates or additives were included in a decay factor preceding transport terms in the species transport equation as presented in the recent study.^38^ Values for the adsorption energies of sulfur and lithium sulfides by PEDOT:PSS and BNG were taken from past studies.^38,44^ The adsorption energies of sulfur and lithium sulfides by SF were predicted in the present study, employing the Blends module of Materials Studio 6.1 (Accelrys, US) and considering a small fragment of SF. Reaction kinetics parameters were taken from our past study^22^ with the exception of electrochemical reactions occurring in the BNG interlayer, where the standard potentials for the conversion reactions to Li_2_S_2_ and Li_2_S were increased to U^o^_Li2S4→Li2S2_ = 2.03 V and U^o^_Li2S2→Li2S_ = 1.96 V (from U^o^_Li2S4→Li2S2_ = 1.87 V and U^o^_Li2S2→Li2S_ = 1.86 V in the rest of the KB-based cathode).

The finite volume/finite difference method was employed for the solution of the numerical equations.^21,59^ The cell comprised a cathode coating with a thickness of 200 μm (measured as the coating thickness after drying in cathode coating fabrication), a BNG interlayer with a thickness of 3 μm, and a separator with a thickness of 25 μm. A one-dimensional grid of 146 points was constructed along the cell thickness, the first point being on the anode surface, which was also the anode-separator interface, followed by 9 points in the separator, 3 points in the BNG interlayer, and 133 points in the cathode.

## 3 Results and Discussion

### 3.1 Experimental Studies

**Figure 2:**
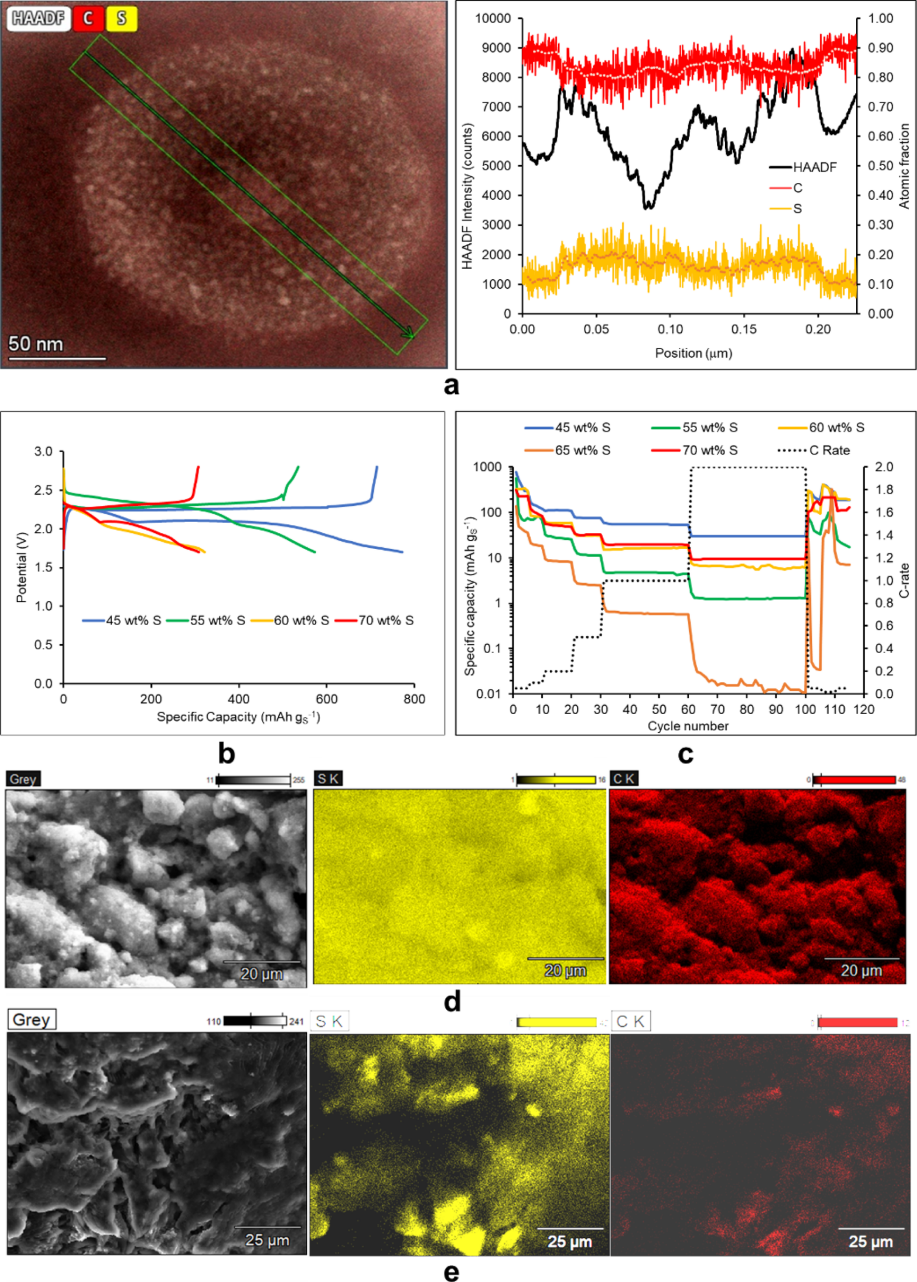
Li–S cells with a KB-based cathode (and PEDOT:PSS binder): (a) STEM/EDX mixed element map of C (red) and S (yellow) of an agglomerate of sulfur infiltrated KB nanoparticles at 39:61 g/g KB:S ratio, with graph depicting the carbon and sulfur atomic fractions comprising the EDX signal across the green-bordered area scanning in the direction of the green arrow. (b) The experimental data of the first galvanostatic discharge–charge cycle at 0.05 C of Li–S cells with cathodes with different sulfur content, from 45 to 70 wt % S. (c) Experimental data of the specific capacity at discharge vs cycle number for a cycling schedule of the Li–S cells at different C-rates. (d, e) SEM image and EDX C and S element maps of the as-fabricated cathode (d) and postmortem (e) after the cycling schedule in (c) for the Li–S cell with 45 wt % S in the KB-based cathode.

Figure 2 presents the experimental data from the testing and characterization of Li–S cells with KB-based cathode coating, including 10 wt % PEDOT:PSS binder, and different contents of sulfur. Figure 2a presents the HAADF-STEM image and mixed element (S and C) EDX map of a particle agglomerate from the sulfur-infiltrated KB via the melt/vapor infiltration method at an S:KB ratio of 61:39 g/g (equivalent to 55 wt % in the final cathode coating with 10 wt % PEDOT:PSS), which shows that the infiltrated sulfur binds the KB particles of outer diameter of about 30 nm^21^ to spherical agglomerates of about 200 nm. EDX analysis and scanning along a slice diametrically through the S-KB agglomerate show the S and C atomic fraction profiles along this slice in Figure 2a. These profiles, also in association with the HAADF-STEM/mixed element EDX map image, show that sulfur has fully infiltrated the wall pores of the particles. There is good correspondence between the S maxima and the C valleys (and vice versa), demonstrating the filling of the hollow KB particle core (80% of the particle volume, 22 nm core diameter) with sulfur. This means that there is no free volume to accommodate the sulfur expansion. Hence, sulfur was infiltrated via the melt/vapor method only up to an equivalent of 45 wt % S in the final coating. Any additional sulfur was added in the form of Li_2_S_8_ solution, expected to reach only interparticle and interagglomerate gaps. This applies to the cathodes with sulfur content of 55, 60, 65, and 70 wt % in the Li–S cells of Figure 2b,c.

Figure 2b,c presents the results of electrochemical testing of the Li–S battery cells with a KB-based cathode (with 10 wt % PEDOT:PSS binder) and different amounts of sulfur content. The cell with 45 wt % S in the cathode exhibits the highest specific capacity at discharge of 772 mAh g_S_^–1^ observed in the first galvanostatic discharge–charge (GDC) cycle (Figure 2b). Contributions from the pseudocapacitance of PEDOT:PSS are encountered in the first discharge for cell potential below 1.8 V, adding about 100 mAh g_S_^–1^ for the cell with 45 wt % S and 50 mAh g_S_^–1^ for the cell with 70 wt % S. Despite the lower initial capacity of the cells with high S content (320 mAh g_S_^–1^ for the cells with 60 and 70 wt % S), these cells recover to even higher capacity after the applied cycling schedule to 370–410 mAh g_S_^–1^ after 100 cycles (Figure 2c), which is similar to the specific capacity of the 45 wt % S cell after 100 cycles. Figure 2c also exhibits the effect of the C-rate: The 45 wt % S cell demonstrates the best performance at higher C-rates. While the C-rate performance worsens as the S content in the cathode increases from 45 to 55 wt %, it gradually improves while it increases further to 60 and 70 wt%, although it is still much lower than the C-rate performance of the 45 wt % S cathode. Furthermore, the equivalent series resistance (ESR) was determined from the experimental data at the start of each discharge in the GDC cycling schedule of Figure 2c and is presented in Figure S1. Figure S1 shows that ESR is reduced with increasing sulfur content in the cathode. This is attributed to the corresponding higher amount of electrolyte, where while maintaining a constant E/S ratio of 11 μL mg_S_^–1^, the higher sulfur content, which also fills a greater fraction of available pore volume of the porous cathode host and separator, is translated to more added electrolyte. Ultimately, this means that the higher amount of electrolyte fills even less pore volume and wets the porous cell components better, resulting in lower ESR for the higher sulfur cells. It is also noticed that the Li–S cell with 45 wt % S in the cathode displays a 10 times rise in ESR at low C-rates. Simulation of the first GDC cycle at 0.05 C for this cell using our multipore continuum model predicted a certain amount of sulfide migration within the cathode from the current collector side to the cathode surface facing the separator, accumulating in the micro- and mesopores of the porous KB particles.^38^ The postmortem SEM image and the EDX S and C element maps in Figure 2e compared with similar images of the as-fabricated cathode in Figure 2d confirm this prediction, in fact exhibiting postmortem deposits around the KB agglomerates on the cathode surface, having smoothened the more porous texture displayed in the SEM of Figure 2d, coupled with very high S element concentration in the S element map in these agglomerates on the cathode surface.

Figure 2c shows that all cells generally exhibit low specific capacity when tested at the 2 C rate. For example, the Li–S cell with 55 wt % S in the cathode starts with an ESR of 100 ohms at 0.05 C, which falls to a minimum of 50 ohms at 2 C after 100 cycles. With such an ESR value at the high rate of 2 C, the cell potential falls immediately under 2 V at the first point of discharge, which means that there is little chance for the redox reactions corresponding to the high voltage plateau of Li–S batteries. In the absence of any formed mid-order sulfide Li_2_S_4_, no reactions can then take place at the low-order plateau either. Hence, the cell potential falls immediately below the minimum set discharge voltage for this cell at 2 C. Furthermore, the 2 C current densities for the different sulfur content cells in this study were in the range of 3–11 mA cm^–2^, which are at very high risk of dendrite formation at the anode according to Sand’s equation.^36^ For this reason, 2 C GDC testing was eliminated from the schedule of subsequent electrochemical testing of Li–S battery cells in this study.

Delving a little more into the effect of sulfur content in the cathode on the specific capacity and cyclability of Li–S cells in Figure 2b,c, it seems that there is a fluctuating trend in the cycling performance of cells with sulfur content in the range of 55–70 wt %. For these cathodes, the first 45 wt % S was infiltrated following the melt/vapor route, and it is expected that most of the sulfur impregnated the core and the microporous wall of the hollow KB particles. The rest of the sulfur, 10–25 wt %, was infiltrated in the form of Li_2_S_8_ via the solution route, and it is expected that it occupied the mesopores between the KB particles. In such a case, the produced soluble sulfides would easily migrate from such large pores to the anode, especially in the case of the lower sulfur content, e.g., 55 wt %, where the mesopores would be only partially filled and still leave unfilled pore space for the soluble sulfides to move. This would reduce the specific capacity and the cyclability of the Li–S cell. As the sulfur content is increased to 70 wt %, the mesopores are filled to a greater extent, and the sulfides cannot move so freely. Sulfide migration is reduced as cyclability improves, as seen in Figure 2c for the 70 wt % S cathode. However, the high sulfur concentration may create a semisolid state and concentration overpotential, reducing the specific capacity at least in the first cycle(s), until Li^+^ ions find a route to access the active cathode material. Concentration variations in the coating may shift the balance of these counteracting mechanisms and may explain the fluctuation in the cycling performance for the Li–S cells with sulfur content in the range of 55–65 wt %. Other factors affecting this fluctuating trend are the thermodynamics of phase equilibrium between the solid and dissolved state of the multiple coexisting sulfides and sulfur during discharge and charge,^60^ weakly dissolved sulfur and sulfides at locally high concentrations of these species in the mesopores,^61^ and the effect of TFSI^–^, depending on its local concentration, on the solvation of sulfides and local regulation of redox reaction kinetics.^62^

The next design step is to evaluate Li–S cells with a BNG interlayer. A preliminary set of experiments with a commercial Nanomyte sulfur cathode (70 wt % sulfur in a carbon black coating with PVDF binder) included: a sprayed BNG interlayer on the cathode surface only; a sprayed BNG interlayer on the separator surface only (the surface facing the cathode); and finally, BNG interlayers sprayed on both the cathode surface and the separator surface. The results of electrochemical testing presented in Figure S2 demonstrate that the Li–S cell with the BNG interlayer sprayed on the cathode surface has the highest specific capacity at discharge and a certainly lower amount of BNG than the cell with two BNG interlayers on both the cathode and separator. The specific capacity of the cell with the BNG interlayer on the cathode surface is higher by 42% than the specific capacity of the cell with the plain Nanomyte cathode (without any BNG interlayer). This is much better progress than that reported in the literature for an interlayer of B,N nanosheets and graphene (only 10% rise),^45^ which is attributed to multiple mechanisms: The high adsorption energies of the BNG interlayer^44^ can prevent sulfur and sulfides from leaving the cathode, while their good conductivity and direct contact with the cathode ensure good electron transport from the carbon cathode host across the BNG interlayer to the sulfur and sulfides trapped there, so they remain active in the Li–S battery redox reaction chain.

Investigations then proceeded to the high-performance KB-10 wt % PEDOT:PSS cathode with a very thin BNG interlayer (with 10 wt % PEO binder) on the cathode surface. Three different E/S ratios were assessed: E/S = 16, 11, or 8 μL mg_S_^–1^. Figure S3a,b display the results of the first GDC cycle and the cycling performance, respectively. Both figures demonstrate that the optimum electrolyte content is at E/S = 11 μL mg_S_^–1^. The other two E/S ratios display a lower and uneven discharge curve at the low voltage plateau than the optimum E/S ratio discharge curve, indicating a lack of Li^+^ ions to carry on with the redox reactions, which is attributed to poor wetting of the cathode pores and low Li^+^ ion transport rate. While this is understandable for the low E/S ratio of 8 μL/mg_S_, it is thought that at the high E/S ratio of 16 μL/mg_S_ the cell becomes overflooded initially, which leads to outward electrolyte flow to the peripheral channel between the electrode discs of 19.2 mm diameter and the cell cavity of 25 mm diameter, and this leaves the main cell poorly wetted. Hence, the rest of the cells with 45 wt % S in the cathode were fabricated with the optimum E/S = 11 μL/mg_S_.

**Figure 3:**
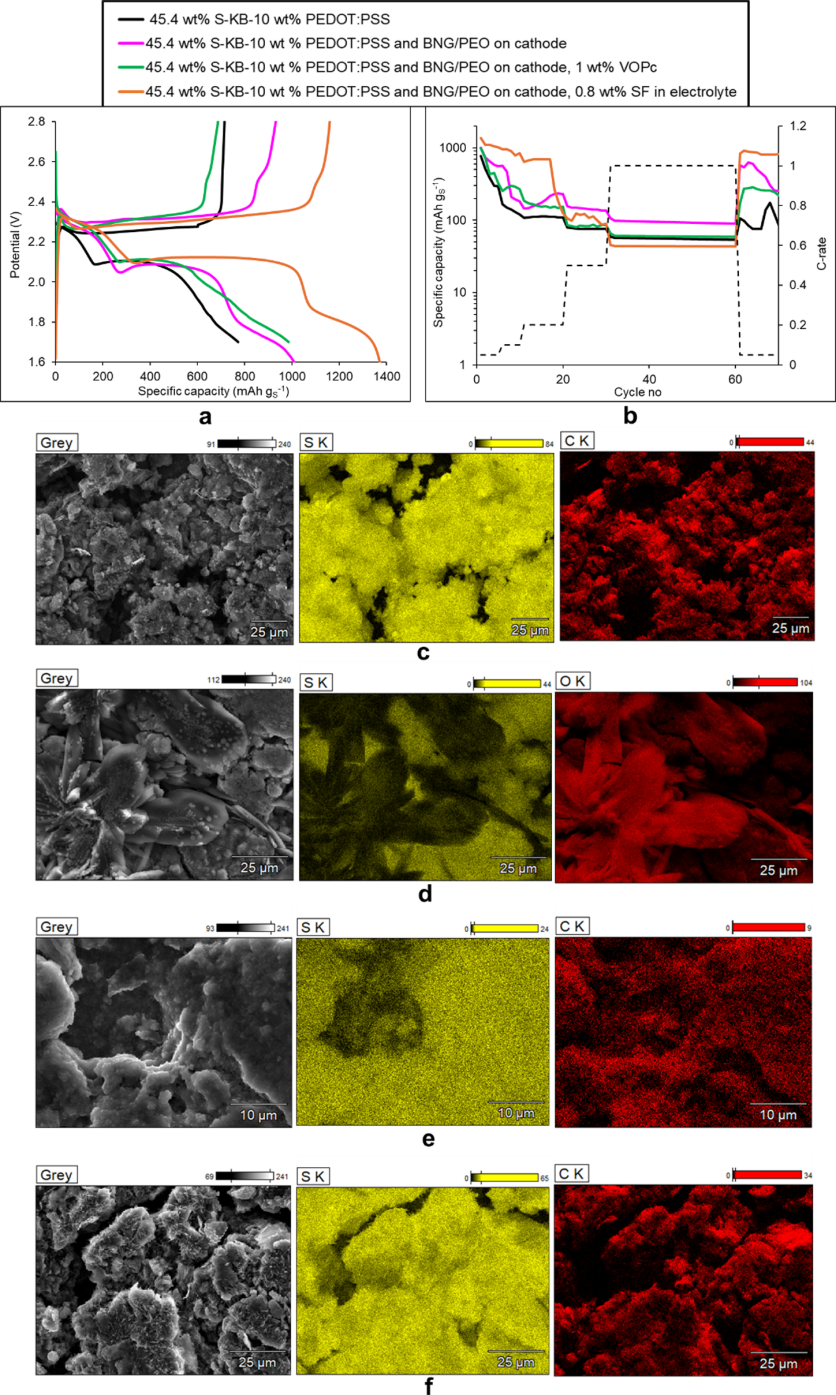
Experimental data for Li–S cells with 45 wt % S-KB-10 wt % PEDOT:PSS cathode and other additives according to the four design steps, as labeled in each subfigure. (a) First GDC cycle at 0.05 C. (b) Specific capacity at discharge versus cycle number for a cycling schedule of the Li–S cells in a schedule of different C-rates. (c–f) SEM image and EDX element maps of (c) as fabricated cathode and (d–f) postmortem cathode after cell cycling.

Figure 3a,b presents the electrochemical test data from the first GDC cycle and the cycling schedule at different C-rates of cells with 45 wt % S in the cathode, designed according to the four strategy steps presented in Figure 1. Figure 3 demonstrates that the BNG interlayer on the cathode improves the specific capacity by at least 10% in the first discharge (Figure 3a) and by 260% in the end part of the cycling schedule (Figure 3b) proving without doubt the beneficial role of the thin BNG interlayer on the cathode surface in Li–S battery cyclability and energy density performance. Furthermore, BNG has potential for also acting as an electrocatalyst for these reactions^46,47^ in the format of a heterogeneous promoter.^63^ The ESR profile at the start of discharge during the GDC cycling schedule for the Li–S cell with the BNG interlayer on the cathode of the 45 wt % S-KB-10 wt % PEDOT:PSS is at a lower level than that of the cell without the BNG interlayer, without the high ESR values of the latter at low C-rate and half the ESR values of the latter at 1C (Figure S4). This is attributed to the higher amount of liquid electrolyte added in the former cell, E/S = 16 μL mg_S_^–1^, against E/S = 11 μL mg_S_^–1^ in the latter.

Subsequently, a moderately conducting network of electrocatalyst VOPc or CoPc molecules was created in the catholyte to add mobile promoters in the mesopores.^63^ The addition of VOPc in the catholyte of Li–S cells with a 45 wt % S-KB-10 wt % PEDOT:PSS cathode and a BNG interlayer on the cathode surface did not add significant benefits. Figure 3a shows that the VOPc brings a small improvement in the specific capacity in the first discharge. The experimental data from the GDC cycling schedule in Figure 3b depict the Li–S cell with 1 wt % VOPc overtaking the rest of the cells reported so far in the 5 cycles at 0.1 C in the first part of the cycling schedule. However, as the C-rate is further increased, the cell with the cathode with just the BNG interlayer overtakes the rest of the cells (reported so far) and retains its good performance in the last part of the schedule at low C-rate. The ESR profiles during the cycling schedule in Figure S4 show that ESR falls with cycling and is maintained at relatively low values during the whole cycling schedule for the cell with VOPc additive in the catholyte. It must be mentioned that a cell with 1 wt % CoPc in the catholyte, while starting with a similar first discharge profile as the cell with 1 wt % VOPc in the catholyte, failed abruptly at 400 mAh/g_S_ which was attributed to short-circuiting of the cell due to CoPc migration across the separator to the anode and the much higher electrical conductivity of the CoPc network compared with VOPc.^52−54^

Finally, the addition of silk fibroin (SF) in the electrolyte brings an impressive increase in specific capacity to 1372 mAh g_S_^–1^ at first discharge (Figure 3a) and the best cycling performance of all cells (Figure 3b). However, Figure S4 shows that the presence of 0.8 wt % SF in the electrolyte increases the cell resistance, which would somehow limit cell performance at high C rates. This increase in cell resistance in the presence of SF is confirmed by another experimental study of Li–S cells.^56^

Figure S5 presents GDC plots in the 100th cycle of a cycling schedule as in Figure 3a for each of the above cells and demonstrates that the cell with the BNG interlayer on the cathode and 0.8 wt % SF in the electrolyte maintains the highest discharge capacity at 920 mAh g_S_^–1^, followed by the cell with just the BNG interlayer on the 45 wt % S-KB-10 wt % PEDOT:PSS cathode that maintains 635 mAh g_S_^–1^. With regard to cell overpotential, the potential difference between charge and discharge at capacity midpoint is consistently low for the Li–S cell with 0.8 wt % SF in the electrolyte (1st cycle in Figure 3a and 100th cycle in Figure S5) at about 0.2 V. It seems that despite the expected electrocatalyst role for the VOPc additives in the catholyte, as predicted from the Gibb’s free energy plots and activation energies predicted by DFT simulations,^47^ this is not translated to overall overpotential reduction (Figures 3a and S5). This is attributed to the higher kinetic overpotential^64^ because of charge transfer limitations across the VOPc network in the mesopores due to the significantly lower conductivity of these semiconductors^52−54^ compared to that of the carbon host. Additionally, these semiconductor materials add their own open circuit voltage, *V*_oc_, reportedly directly related to their first reduction potential,^65^ which is 0.6 V vs. RHE for CoPc^66^ while the *V*_oc_ determined from the HOMO–LUMO energy gap^67^ is 1.94 V for CoPc^68^ and 0.9 V for VOPc.^69^

Figure S6 presents SEM images of the as-fabricated cathode with sprayed BNG on the cathode surface, where exfoliated BNG flakes of 2–5 μm lateral dimensions can be observed to have formed a layer of one or two flake depths, sometimes overlapping at the edges. The EDX element maps of B and N in Figures S6 and 3c present a good coverage of the cathode surface coinciding with the C element map. The S element map is more expanded, representing not just sulfur on the cathode surface but also through a few microns depth. The postmortem EDX element maps in Figure 3, after the full cycling schedule in Figure 3b, present S and C deposits from the LiTFSI salt, as for the cell with the 45 wt % S-KB-10 wt % PEDOT:PSS cathode in Figure 3d, S fully distributed in low intensity due to its attachment to the VOPc in the catholyte in Figure 3e, and in high intensity well attached to the SF electrolyte additive in Figure 3f.

**Figure 4:**
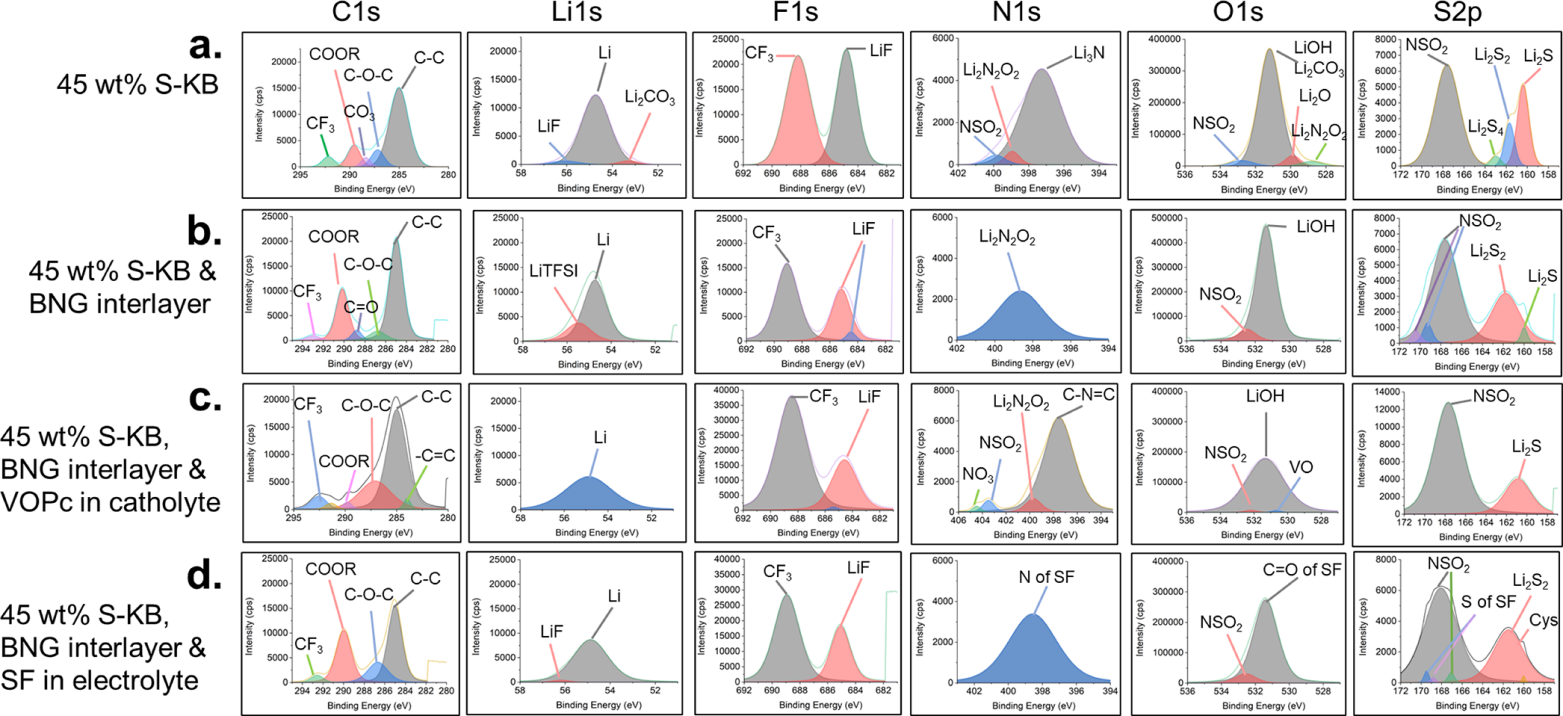
Post-mortem high-resolution XPS spectra of the anodes of the following Li–S cells: (a) Cell with a cathode coating of 45 wt % S in KB and 10 wt % PEDOT:PSS binder. (b) Cell with a cathode coating of 45 wt % S in KB and 10 wt % PEDOT:PSS binder, with a BNG interlayer sprayed on the cathode surface. (c) Cell with a cathode coating of 45 wt % S in KB and 10 wt % PEDOT:PSS binder with a BNG interlayer sprayed on the cathode surface, and 1% VOPc added in the catholyte. (d) Cell with cathode coating of 45 wt % S in KB with 10 wt % PEDOT:PSS binder and BNG interlayer sprayed on the cathode surface, as well as 0.8 wt % SF (silk fibroin) in the electrolyte.

Figure 4 presents the full high-resolution XPS spectra of the anodes of the different Li–S cells postmortem after their cycling schedule. Figure 4a with the plain 45 wt % S-KB cathode and 10 wt % PEDOT:PSS binder, shows that an SEI (solid-electrolyte interface) was formed on the Li anode consisting of LiF, Li_2_CO_3_, Li_2_N_2_O_2_, Li_3_N, LiOH, and Li_2_O. It also indicates the presence of LiTFSI electrolyte salt. Additionally, the S 2p spectrum exhibits a high-intensity peak indicating the presence of Li_2_S and smaller peaks indicating the presence of Li_2_S_2_ and Li_2_S_4_ that migrated and precipitated at the anode. Figure 4b with a 45 wt % S-KB cathode (and 10 wt % PEDOT:PSS binder) and BNG interlayer on the cathode surface, shows that a thinner SEI was formed on the Li anode than in Figure 4a, consisting of LiF (but half the amount encountered in Figure 4a), Li_2_N_2_O_2_ and LiOH. The S 2p spectrum indicates the presence of Li_2_S_2_ and Li_2_S but in smaller amounts than the total amount of sulfides in Figure 4a, confirming that the BNG interlayer reduces the migration of sulfides to the anode. Figure 4c with a 45 wt % S-KB cathode (and 10 wt % PEDOT:PSS binder), BNG interlayer on the cathode surface, and VOPc in the catholyte, displays evidence of sp^2^ carbon in the C 1s spectrum of the anode, which originates from VOPc, indicating that VOPc has traveled from the catholyte, traversed the separator, and reached the anode surface. The N 1s spectrum also displays a peak related to the −C—N=C— part of VOPc. There is a thin SEI on the Li anode surface consisting of LiF (but less than in Figure 4a), Li_2_N_2_O_2_ and LiOH. The S 2p spectrum reveals some Li_2_S on the anode but at a smaller percentage than the sulfides on the anode in Figure 4a,b. This means that the VOPc added in the catholyte traps even more sulfides, although some VOPc has also migrated to the anode. Figure 4d with a 45 wt % S-KB cathode (and 10 wt % PEDOT:PSS binder), BNG interlayer on the cathode surface, and silk fibroin (SF) in the electrolyte, displays evidence of an SF layer coating the anode as seen from additional SF-related peaks^70,71^ in the N 1s, O 1s, and S 2p spectra, where the latter also divulges the presence of cysteine in SF.^72,73^ Apart from the expected presence of LiTFSI electrolyte salt, there is still the presence of LiF on the Li anode but no other byproducts of parasitic reactions as the multi-component SEI formed on the anode in Figure 4a and even Figure 4b,c. The S 2p spectrum displays a peak associated with Li_2_S_2_ that also contains a subpeak associated with the sulfur of cysteine (Cys) present in SF.^72^

The postmortem SEM images of the anode of the tested cells in Figure S7 show clear evidence of bundles of ribbons and dendrites for the cells in Figure S6a–d. Figure S6e demonstrates that the silk fibroin has smoothed the surface roughness significantly, showing that it can suppress the aggressive growth of dendrites.

A higher sulfur concentration of 55 wt % in the cathode leads to a similar specific capacity at first discharge of 930 mAh g_S_^–1^ for the cell with the BNG interlayer on the cathode (Figure S9a) compared to the corresponding cell with 45 wt % S (Figure 3a) but it falls to 800 mAh g_S_^–1^ for the cell with the BNG interlayer on the cathode and VOPc in the catholyte compared to 978 mAh g_S_^–1^ for the corresponding cell with 45 wt % S at first discharge. However, after the cycling schedule presented in Figure S9b, the discharge of the 100th cycle presented in Figure S9c yields a capacity of only 233 mAh g_S_^–1^ for the 55 wt % cell with the BNG interlayer on the cathode and 585 mAh g_S_^–1^ for the cell with the BNG interlayer on the cathode and the VOPc in the catholyte compared with 633 mAh g_S_^–1^ and 284 mAh g_S_^–1^, respectively, for the corresponding 45 wt % S cells (Figure S5).

### 3.2 Multiscale Simulations

Simulations of the first GDC cycle were conducted employing the digital twin based on the multipore continuum model^21^ to elucidate the physicochemical processes controlling the performance of the major and best-performing types of the Li–S cells explored in this study. The predicted GDC curves for the first GDC cycle are presented in Figure S13a also in comparison with the corresponding experimental data in the same plot. The good agreement between predictions and experimental data encourages the next step of the analysis of the predicted concentration distributions of sulfur and sulfides through the cell in order to identify the key phenomena governing the Li–S cell capacity and energy density. Taking into account also the predicted distribution of species concentrations in our recent study^38^ for a Li–S cell with a cathode of 45.4 wt % S-KB-10 wt % PVDF, the replacement of the PVDF with the PEDOT:PSS binder in the cathode has brought a big drop in the migration of all sulfides and sulfur to the anode, as shown in Figure S14, due to the strong adsorption of these species by the PEDOT:PSS coating the walls of meso and macropores in the cathode. The pseudocapacitance of PEDOT:PSS also extends the specific capacitance by 7–9% due to the surface redox effect of PEDOT:PSS with respect to the Li^+^ ions, evident below 1.8 V in the discharge curve at 0.05 C. Significant internal migration of the soluble sulfides is still predicted in the cathode, from the current collector to the separator, although it is greatly reduced from the extent of migration predicted for the Li–S cell with PVDF binder in the cathode.^38^ The postmortem photo of the anode after the full cycling schedule for the cell with the PEDOT:PSS binder in the cathode, as inserted in Figure S14 (also depicted in Figure S10) displays yellowish and dark brown areas on the anode surface, respectively indicating the presence of sulfur and high-order sulfides, such as Li_2_S_8_ and Li_2_S_6_.^57,58^Figure S14 exhibits a very small amount of Li_2_S_6_ on the anode surface, 7.4 mol m^–3^, after the first discharge, that may accumulate and rise after 100 cycles at different C-rates, which would explain the presence of the dark brown deposit on the anode surface in the postmortem photo.

**Figure 5:**
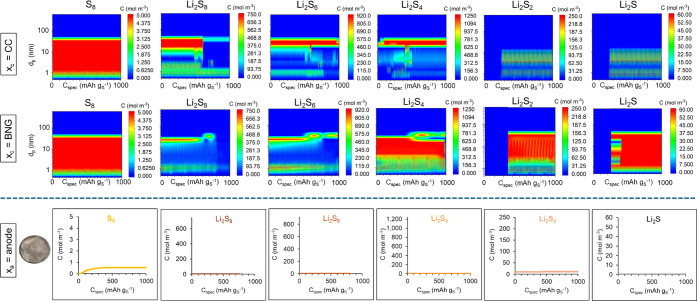
Predicted concentrations of the dissolved sulfur and sulfides in the liquid electrolyte as a function of specific capacity during the first discharge of the Li–S battery cell with cathode of 45 wt % S-45.4 wt % KB–10 wt % PEDOT:PSS coating and a thin BNG interlayer on the cathode surface: contour plots in cathode as a function of pore size for two different locations (by the current collector and in the BNG interlayer) and concentration profiles at anode, together with a postmortem photo of the anode after the full cycling schedule. The maximum concentration limit in each plot is set to the saturation concentration of that species in the electrolyte solution.

Figure 5 presents the predicted contours and profiles of species concentrations after the first discharge of the Li–S cell with a cathode of 45.4 wt % S-KB-10 wt % PEDOT:PSS sprayed with a thin BNG interlayer. In comparison with Figure S14, it can be seen that the BNG interlayer coating the cathode surface has substantially slowed the transport of the high-order sulfides, Li_2_S_8_ and Li_2_S_6_, through the cathode from the current collector to the cathode surface, which prolongs the redox reactions and extends the specific capacity of the cathode.

Furthermore, in order to optimize the fitting of the predicted GDC curves with the experimental data, the standard potential of the last two conversion reactions was increased to U^o^_Li2S4→Li2S2_ = 2.03 V and U^o^_Li2S2→Li2S_ = 1.96 V (from U^o^_Li2S4→Li2S2_ = 1.87 V and U^o^_Li2S2→Li2S_ = 1.86 V in the rest of the KB-based cathode). Although the predicted migration of sulfur and sulfides Li_2_S_8_, Li_2_S_6_, and Li_2_S_4_ is kept to a minimum (Figure 5), the amount of Li_2_S_2_ on the anode surface has increased, which is attributed to the larger amount of Li_2_S_2_ produced in the cathode and BNG interlayer in this higher specific capacity cell compared to the cell without a BNG interlayer. This is confirmed by the light brown-yellowish deposit on the anode surface depicted in the postmortem photo inserted in Figure 5, indicating the presence of Li_2_S_2_ and S_8_.

**Figure 6:**
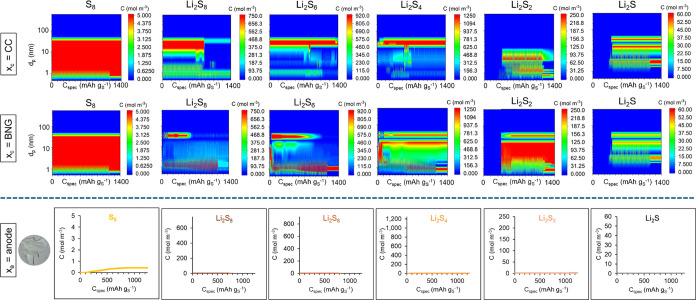
Predicted concentrations of the dissolved sulfur and sulfides in the liquid electrolyte as a function of specific capacity during the first discharge of the Li–S battery cell with cathode of 45 wt % S-45.4 wt % KB–10 wt % PEDOT:PSS coating and a thin BNG interlayer on the cathode surface as well as 0.8 wt % SF in the electrolyte: contour plots in cathode as a function of pore size for two different locations (by the current collector and in the BNG interlayer) and concentration profiles at anode, together with a postmortem photo of the anode after the full cycling schedule. The maximum concentration limit in each plot is set to the saturation concentration of that species in the electrolyte solution.

Figure 6 presents the predicted contours and profiles of species concentrations after the first discharge of the Li–S cell with a cathode of 45.4 wt % S-KB-10 wt % PEDOT:PSS sprayed with a thin BNG interlayer and also with 0.8 wt % SF in the liquid electrolyte. In comparison with Figures 5 and S14, the predicted migration of all species is reduced both through the cathode and to the anode, which explains the highest specific capacity of this type of cell as it allows the species to react up to the highest degree *in situ* until the expanded species volume reaches the pore volume at the end of discharge. This is attributed to the local adsorption of the sulfur atom-containing species by the silk fibroin molecules present in the pores. These predictions of low sulfur and sulfide migration to the anode are confirmed by the postmortem photo of the anode shown in Figure 6, which shows a clear anode without any yellow or brown discoloration from sulfur or sulfide deposits.

A question arose with regard to the physicochemical model about which pore sizes can accommodate the SF molecule. Molecular modeling of the SF structure solvated in DOL:DME 1:1 v/v yielded an SF diameter of 3 nm, the diameter of the first layer of coordinated DOL solvent being 4 nm, and the diameter of the first layer of coordinated DME being 8 nm.^56^ As there was some uncertainty for the present study about whether the SF structure could deform to be inserted in pores smaller than 3 nm, a parametric study of multipore continuum model-based simulations of the first discharge was conducted with SF in pore sizes d_p_ > 1 nm, d_p_ > 1.5 nm, d_p_ > 2 nm, or d_p_ > 3 nm. The predicted first discharge curves at 0.05 C are presented in Figure S13b yielding specific capacities of 49, 59, 1010, and 1270 mAh g_S_^–1^, respectively, with the last case study of SF in d_p_ > 3 nm fitting the experimental data best. It seems that if SF is present in all pore sizes, its adsorption of locally produced sulfides does not allow any interpore transport, so the volume expansion generated from the early production of Li_2_S_8_ quickly fills the micropores fully with solids without the presence of any liquid electrolyte. Given that the redox reaction chain for electrolyte 1 M LiTFSI in DOL/DME is assumed to take place only in the liquid phase, this stops any further reaction and any further transport of Li^+^ ions through the pores of the KB particle wall to the hollow KB particle core that contains the main part of sulfur, which leads to the rapid depletion of Li^+^ ion supply and ceasing of the redox reaction chain. If the model allows SF in meso- and macropores of d_p_ > 3 nm (where the size of the SF structure is in fact 3 nm),^56^ it seems that the sulfide transport allowed through the micropores accommodates the expansion of the active cathode material over the whole pore size distribution of the cathode host and the continuous presence of the liquid electrolyte in many of these pores, maximizing the extent of redox reactions and the specific capacity.

## 4 Conclusions

A multistep strategy was devised and tested to optimize the performance of Li–S batteries. The evaluation was based on experimental studies of small Li–S cells and multipore continuum model-based simulations with key properties supplied from MD or DFT simulations. Predicted GDC curves for the different types of Li–S cells explored in this study exhibited very good agreement with experimental data, contributing to the validation of our numerical model in the presence of BNG interlayer, heterogeneous and mobile promoters, and traps of soluble sulfides.

The use of a hollow conductive particle-based cathode host was the basis of the strategy, together with the cathode coating binder PEDOT:PSS. Ketjenblack EC-600JD was selected as a commercially available conductive hollow particle powder, which can accommodate up to 45 wt % sulfur in the cathode coating (including 10 wt % binder) impregnated in the hollow particle core and its ultimate expansion upon full conversion to Li_2_S. This cathode was tested with a low E/S ratio of 8–11 μL mg_S_^–1^ and reached 772 mAh g_S_^–1^ at first discharge, falling to 193 mAh g_S_^–1^ at the 100th discharge at 0.05 C after a schedule of cycles at different C-rates. Multipore continuum model-based simulations showed that PEDOT:PSS contributed with its pseudocapacitance by 7–9% to the overall specific capacity of the battery below 1.8 V, which was confirmed by the shape of the experimental discharge curve. Furthermore, the hollow particle host and the PEDOT:PSS binder combined their physical constraining action and strong adsorption energy, respectively, to slow down the sulfur and sulfides migration to the anode. However, a low amount of sulfur, Li_2_S_8_ and Li_2_S_6_ sulfides were still predicted at the anode surface at the end of the first discharge that would accumulate with cycling, deteriorating the battery performance, which was also confirmed by the yellowish and dark brown areas on the anode surface from the postmortem anode photo after the full cycling schedule of this study.

The second successful strategy step was a sprayed thin BNG layer on the cathode surface, with an estimated thickness of about 3 μm from SEM images. This increased the specific capacity to 1010 mAh g_S_^–1^ at the first discharge, falling to 633 mAh g_S_^–1^ at the 100th discharge after a cycling schedule at different C-rates. The higher specific capacity and better cyclability of this type of Li–S cell were attributed to the further retention of sulfur and sulfides by the increased tortuosity of the BNG platelets and the adsorption energy of the B–N- groups, as per the predictions of the multipore continuum model-based simulation of the first GDC cycle. This allowed only a small amount of sulfur and Li_2_S_2_ to migrate to the anode, which was confirmed by the light brown-yellowish area on the anode surface from the postmortem anode photo after the full cycling schedule of this study. The novel BNG interlayer on the cathode surface proposed in this work has the advantage of being in direct contact with the conductive cathode, which ensures excellent electron transfer to any sulfur and sulfides trapped in the BNG interlayer, retaining them active. In fact, they react under favorable conditions in the presence of the B–N– electrocatalyst groups. Li–S cells with a sprayed BNG interlayer on the separator or on both the cathode surface and the separator exhibited inferior performance due to the loss of any sulfur and sulfides trapped at the separator, as they did not receive electrons from the cathode and ceased any participation in the redox reaction chain.

The final successful strategy step was the addition of 0.8 wt % silk fibroin in the liquid electrolyte, which yielded a specific Li–S cell capacity of 1372 mAh g_S_^–1^ at first discharge and 920 mAh g_S_^–1^ at the 100th discharge after a cycling schedule at different C-rates. A multipore continuum model-based simulation of the first cycle predicted further reduction of the sulfide migration to the anode due to their adsorption by the SF additive present in the electrolyte in pores greater than 3 nm, which was confirmed by the clear anode surface from the postmortem anode photo after the full cycling schedule. The protective effect of SF in the reduction of dendrites on the anode was also noted via postmortem SEM imaging of the anode. The main conclusion from the step-by-step strategy devised in this study was that the reduction of sulfur and sulfides’ migration to the anode and the retention of their active character in the redox reaction chain played a key role in maximizing the specific capacity and cyclability of Li–S cells with liquid electrolyte. The multipore continuum model-based simulations demonstrated that allowing a small extent of interpore transport was beneficial in distributing the sulfides in order to allow volume expansion, especially in the micropores.

A step of adding VOPc or CoPc in the catholyte to act as an adsorbent of sulfur and sulfides in the meso- and macropores and as an electrocatalyst of the redox reactions in the cathode did not always yield the expected improvement in Li–S battery performance and cyclability. This failure was attributed to slow charge transfer along the VOPc network due to the relatively low conductivity of the VOPc semiconductor network, while the higher conductivity CoPc migrating to the anode easily caused short-circuiting.

Higher sulfur content than 45 wt % S in the cathode was realized by heat impregnating 45 wt % S and adding the rest of the sulfur in the form of Li_2_S_8_ via the solution/evaporation method, so it may fill the interparticle voids, allowing for expansion upon ultimate conversion to Li_2_S. Li–S cells with 55 to 70 wt % S, KB cathode host, and PEDOT:PSS binder started with lower specific capacity in the first GDC cycle than the 45 wt % S cell, which increased with cycling, reaching a maximum of 408, 329, and 211 mAh g_S_^–1^ in the 100th cycle for the cells with the 60, 55, and 70 wt % S cathode, respectively. Testing cells with 55 wt % S in the cathode, the thin BNG interlayer on the cathode yielded 932 and 233 mAh g_S_^–1^ in the first and 100th discharge, respectively. A cell with 55 wt % S in the cathode, a thin BNG interlayer on the cathode, and 1 wt % VOPc in the catholyte yielded 805 and 586 mAh g_S_^–1^ in the first and 100th discharge, respectively, demonstrating the beneficial role of the VOPc network in the meso- and macropores in this case.

## Supporting Information Available

Includes Figures S1–S14. Additional experimental details and data on Li–S cells based on Nanomyte cathode. Additional data of ESR variation with cycling, effect of E/S ratio, GDC curves from the 100th cycle, SEM images of anode of all cells, additional SEM/EDX images of cathode, example of XPS analysis, experimental data and characterization of cells with 55 wt % S in cathode. Post-mortem photos of cell components of all tested cell types. Results of MD simulations with regards to SF and its interactions with sulfides. Predictions of GDC curves versus experimental data (PDF)

## Abbreviations

CoPc: cobalt phthalocyanine
VOPc: vanadyl phthalocyanine
PEDOT: PSS: poly(3,4-ethylenedioxythiophene):polystyrenesulfonate
PVDF: polyvinylidene fluoride
