# Supplementary material for: A Step-by-Step Design Strategy to Realize High-Performance Lithium–Sulfur Batteries
Source: ACS Appl Energy Mater. 2025 Jan 28;8(3):1492–506. doi: 10.1021/acsaem.4c02457 (PMC11822561; doi:10.1021/acsaem.4c02457)
Supplement: Supplementary file 1 — ae4c02457_si_001.pdf [file ae4c02457_si_001.pdf]

Supporting Information

A Step-by-Step Design Strategy to Realize High-Performance Lithium–Sulfur Batteries

*Matthew J. Dent, Sean Grabe, Steven J. Hinder, Mateus G. Masteghin, James D. Whiting, John F. Watts, Constantina Lekakou**

Centre for Engineering Materials, Faculty of Engineering and Physical Sciences, University of Surrey, Guildford, GU2 7XH, UK

E-mail: C.Lekakou@surrey.ac.uk

1. Experimental Methods

Apart from the KB-based cathode (KB denotes Ketjenblack EC-600JD), an alternative cathode tested in section 2.2 includes the commercial Nanomyte® BE-70E sheet (NEI Corp., USA) including a cathode coating of 70 wt% sublimed sulfur in carbon black. In the reported tests in section 2.2, apart from a standard Li-S cell with Nanomyte® cathode, three more types of Li-S cells were tested. They include: a B,N codoped graphene (BNG) interlayer sprayed on the cathode surface, or on the separator surface facing the cathode, or on both the cathode and separator surface.

2. Electrochemical Test Data

2.1 Li-S Cells with KB-based Cathode and PEDOT:PSS Binder

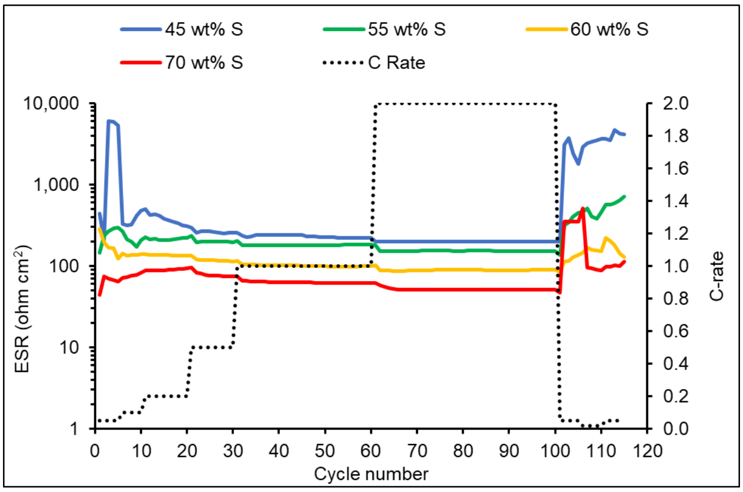

Figure S1. Li-S cells with KB-based cathode (and 10 wt% PEDOT:PSS binder) and different sulfur content: Equivalent-in-series resistance (ESR) determined at start of galvanostatic discharge plotted as ESR profile with respect to the cycle number for a cycling schedule of different C-rates.

2.2 Li-S cells with BNG interlayer

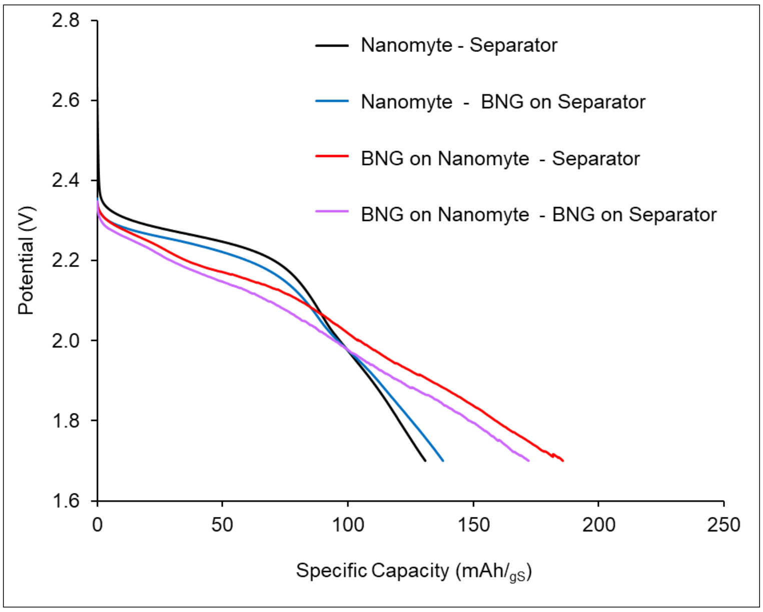

Figure S2. First discharge at 0.05 C of Li-S cells with Nanomyte® cathode. As labeled, three of these cells have BNG interlayer sprayed either on the cathode surface or on the separator surface (facing the cathode) or on both the cathode and the separator surface.

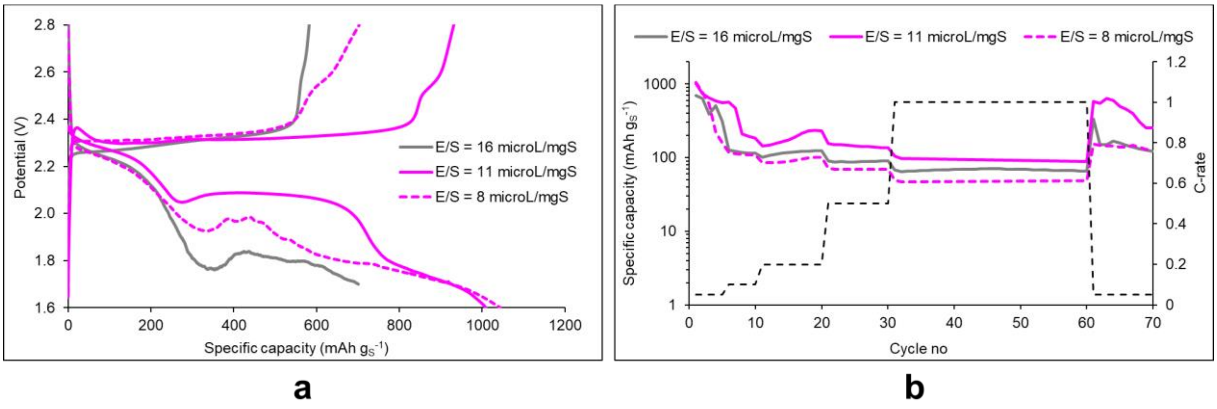

Figure S3. Li-S cells with 45 wt% S-KB-based cathode (and 10 wt% PEDOT:PSS binder) and BNG/PEO interlayer on the cathode at three different E/S ratios: E/S = 16, 11 and 8 μL/mg$_S$. **a.** Results from the first GDC cycle at 0.05 C; **b.** results of the discharge specific capacity as a function of cycle number in a GDC cycling schedule at different C-rates.

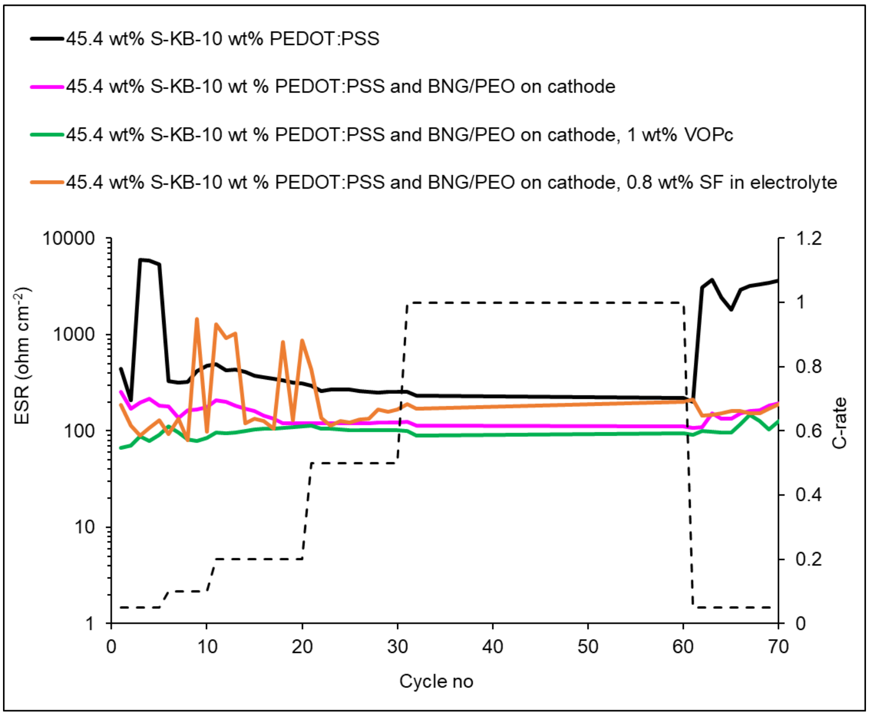

Figure S4. Li-S cells with 45 wt% S-KB-based cathode (and 10 wt% PEDOT:PSS binder) and other additives as labeled on the plot: Equivalent-in-series resistance (ESR) determined at start of galvanostatic discharge plotted as ESR profile with respect to the cycle number for a cycling schedule of different C-rates.

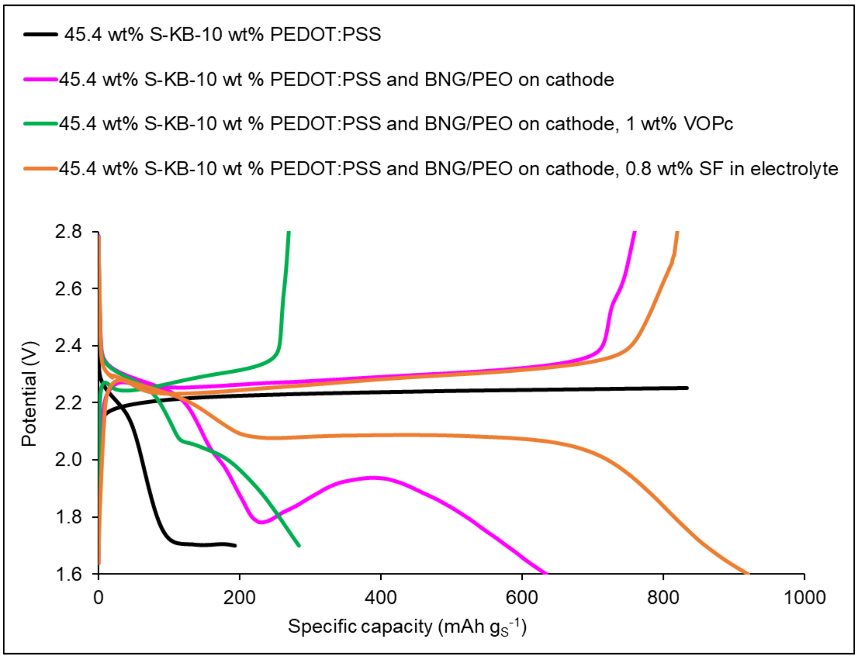

Figure S5. Li-S cells with 45 wt% S-KB-based cathode (and 10 wt% PEDOT:PSS binder) and other additives as labeled on the plot: GDC curves at 0.05 C in the 100th cycle from the GDC cycling schedule at different C-rates (as in Figure S4).

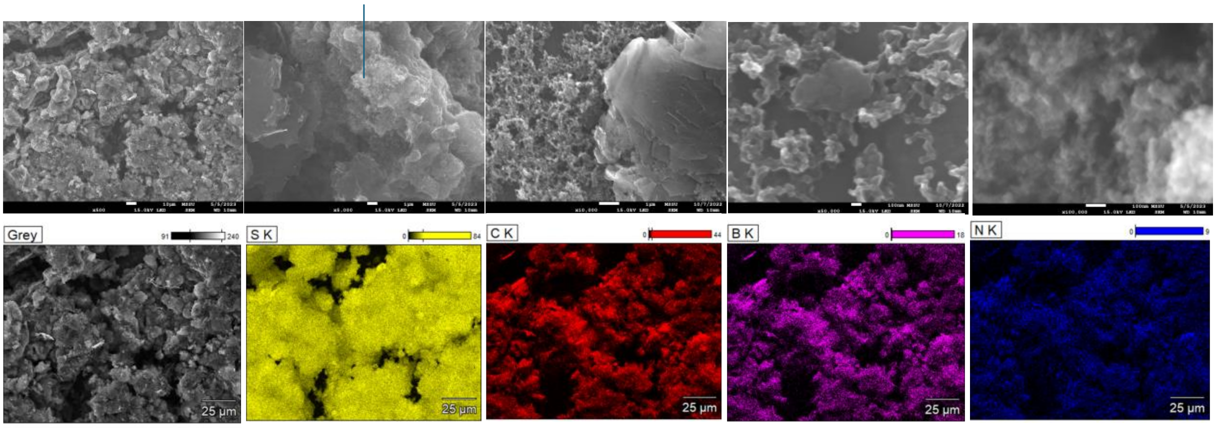

Figure S6. SEM images at different magnification and EDX element maps of the as fabricated cathode of 45.4 wt% S – KB – 10 wt% PEDOT:PSS binder and BNG interlayer sprayed on the cathode surface.

a. 45 wt% S-KB-10 wt% PVDF

b. 45 wt% S-KB-10 wt% PEDOT:PSS

c. 45 wt% S-KB-10 wt% PEDOT:PSS, BNG

d. 45 wt% S-KB-10 wt% PEDOT:PSS, BNG, VOPc

e. 45 wt% S-KB-10 wt% PEDOT:PSS, BNG, SF

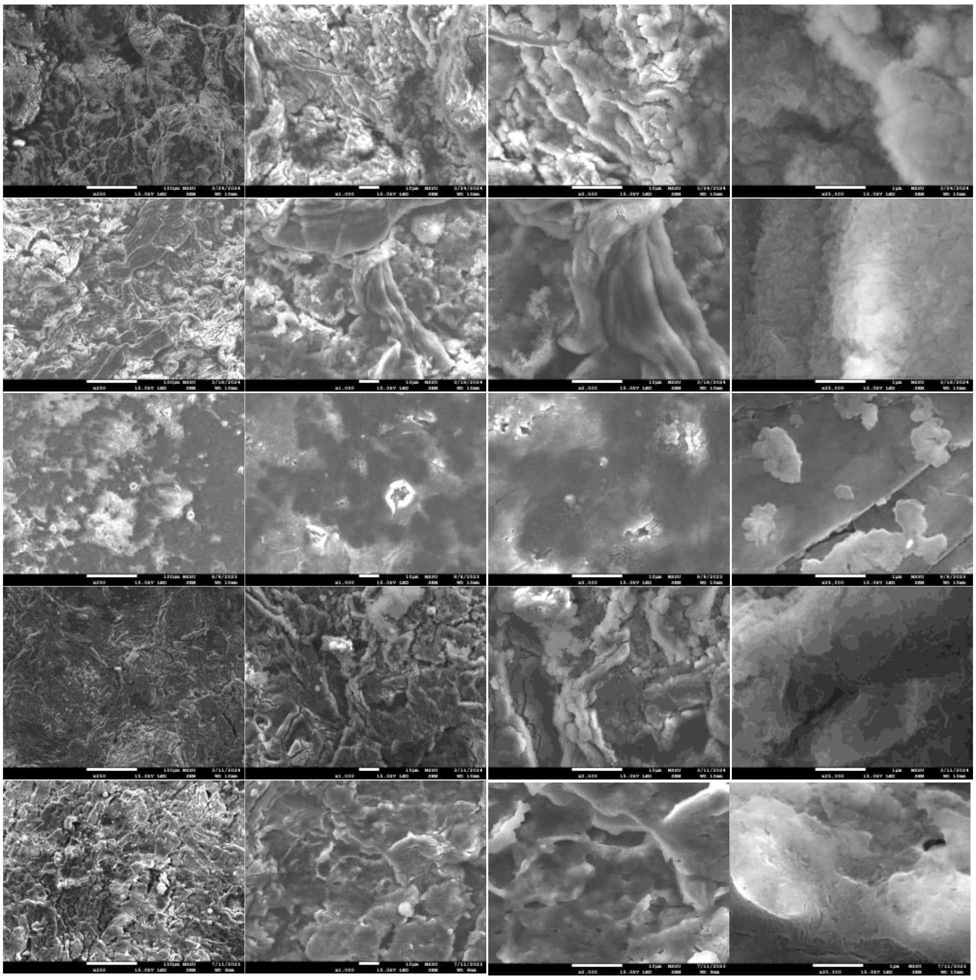

Figure S7. Postmortem SEM images at different magnifications of the anode of Li-S cells as specified in the label of each SEM image row. **a.** Cathode coating is 45 wt% S-KB-10% PVDF; **b-e.** cathode coating is 45 wt% S-KB-10% PEDOT:PSS; **c-e.** also with thin BNG interlayer coating the cathode surface; **d.** also with VOPc in the catholyte; **e.** with silk fibroin (SF) in the electrolyte.

2.3 XPS Analysis of Anodes

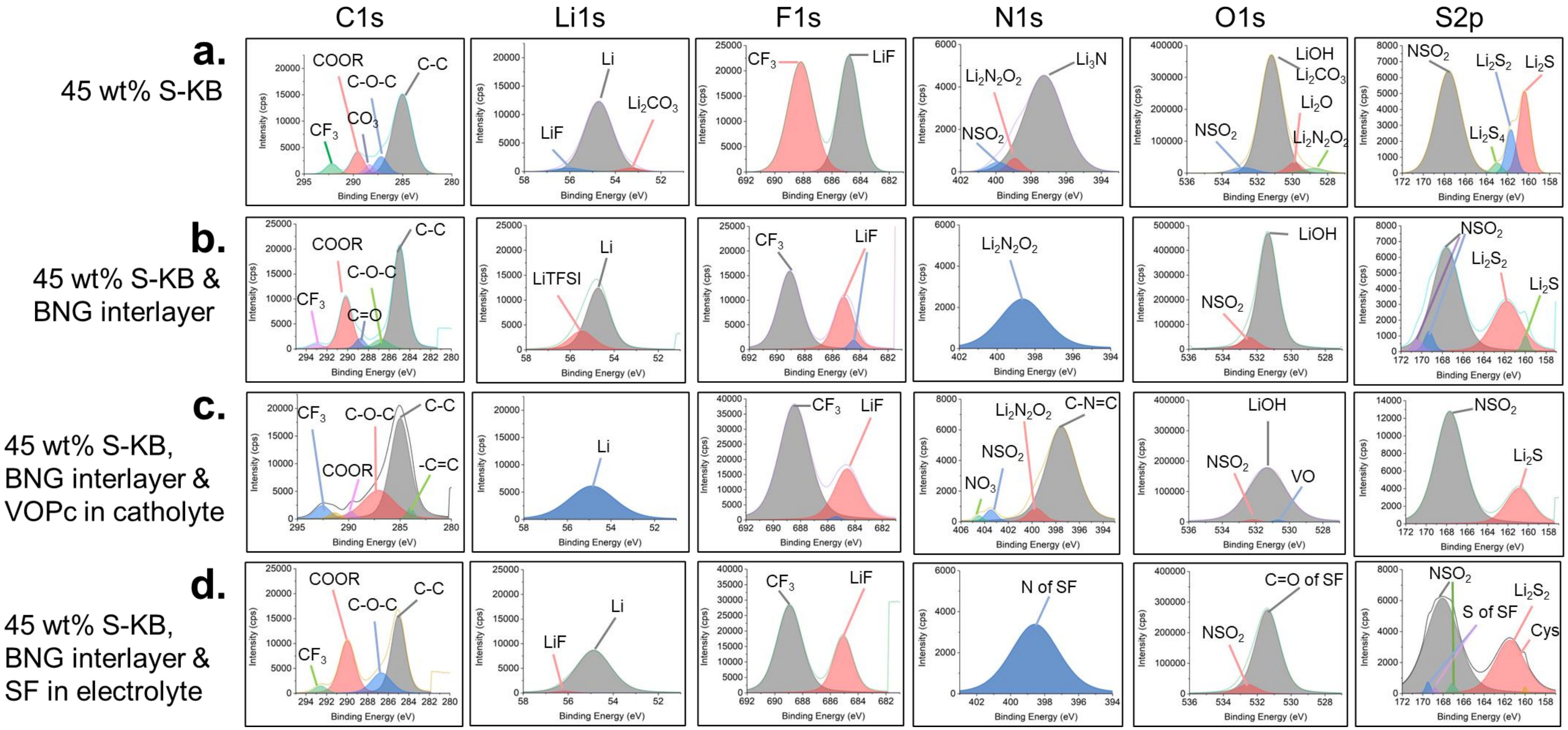

Figure S8. Post-mortem high resolution XPS spectra of the anodes of the following Li-S cells: **a.** Cell with cathode coating of 45 wt% S in KB with 10 wt% PEDOT:PSS binder. **b.** Cell with cathode coating of 45 wt% S in KB with 10 wt% PEDOT:PSS binder and BNG interlayer sprayed on the cathode surface. **c**. Cell with cathode coating of 45 wt% S in KB with 10 wt% PEDOT:PSS binder and BNG interlayer sprayed on the cathode surface, as well as 1% VOPc added in the catholyte. **d**. Cell with cathode coating of 45 wt% S in KB with 10 wt% PEDOT:PSS binder and BNG interlayer sprayed on the cathode surface, as well as 0.8 wt% SF (silk fibroin) in the electrolyte.

Example of XPS analysis:

Figure S7a. 45 wt%S-KB cathode with 10 wt% PEDOT:PSS binder:

The ***C1s spectrum*** comprises a major peak at 285 eV indicating C-C bonds, a peak at 289.6 eV indicating COOR groups, a peak at 287.2 eV indicating C-O-C groups, a peak at 292.2 eV indicating CF_3 groups and a peak at 288.4 eV indicating metal carbonate groups Li_2CO_3 due to parasitic reactions of DOL/DME with the lithium anode.

The ***Li1s spectrum*** comprises a major peak at 54.7 eV associated with Li, a peak at 53.3 eV indicating Li_2CO_3 and a peak at 56.1eV indicating LiF.

The ***F1s spectrum*** comprises two high intensity peaks, a peak at 684.8 eV indicating LiF bonds, a peak at 688.2 eV indicating CF_3 from LiTFSI or other organic fluorine.

The ***N1s spectrum*** comprises a major peak at 397.3 eV indicating metal nitride such as Li_3N, a peak at 398.9 eV indicating $Li_2N_2O_2$ and a peak at 399.9 eV indicating NSO_2 from LiTFSI.

The ***O1s spectrum*** comprises a major peak at 531.2 eV indicating LiOH and Li_2CO_3, a peak at 529.9 eV indicating metal oxides such as Li_2O, a peak at 532.8 eV eV indicating SO_2 groups from LiTFSI and a peak at 528.8 indicating $Li_2N_2O_2$.

The ***S2p spectrum*** comprises a major peak at 167.6 eV SO_2 groups from LiTFSI, a high intensity peak at 160.4 eV indicating Li_2S, a peak at 161.7 eV indicating Li_2S_2 and a peak at 163 eV indicating Li_2S_4.

2.4 Li-S Cells with 55 wt% S-KB-10 wt% PEDOT:PSS and other features

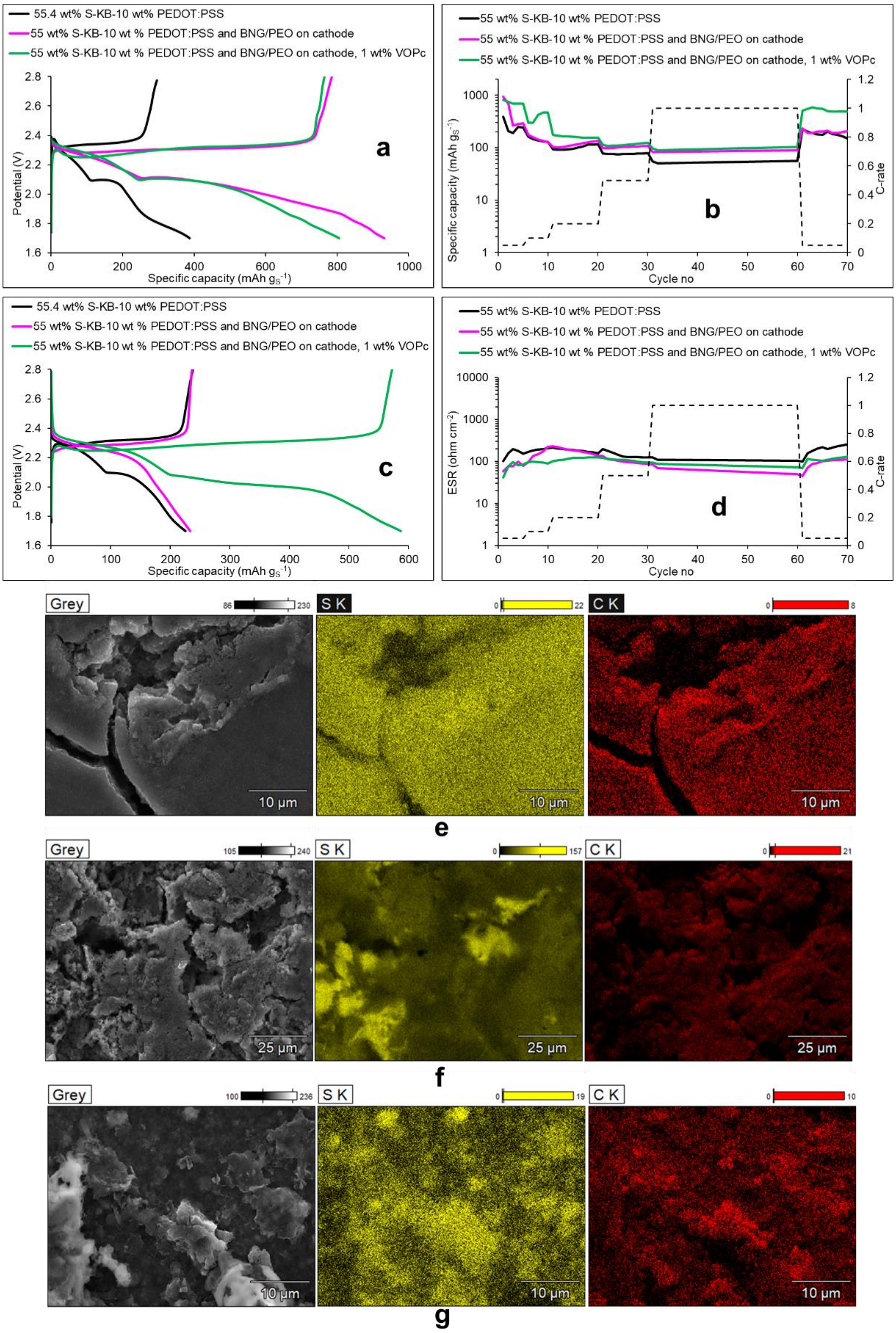

Figure S9. Experimental data for Li-S cells with 55 wt% S-KB-10 wt% PEDOT:PSS cathode and other features **a.** 1st GDC cycle at 0.05 C. **b.** Specific capacity at discharge versus cycle number for a cycling schedule of the Li-S cells at different C-rates. **c.** 100th GDC cycle at 0.05 C. **d.** ESR of cell at start of discharge versus cycle number for the cycling schedule in b. **e-g.** Postmortem SEM image and EDX C and S element maps of the cathode of cells **e.** Cell with cathode of 55 wt% S-KB-10 wt% PEDOT:PSS. **f.** Cell with cathode as in e and BNG interlayer on cathode surface. **g.** Cell with cathode as in f with also VOPc in catholyte.

2.5 Postmortem photos of cell components of all tested cell types

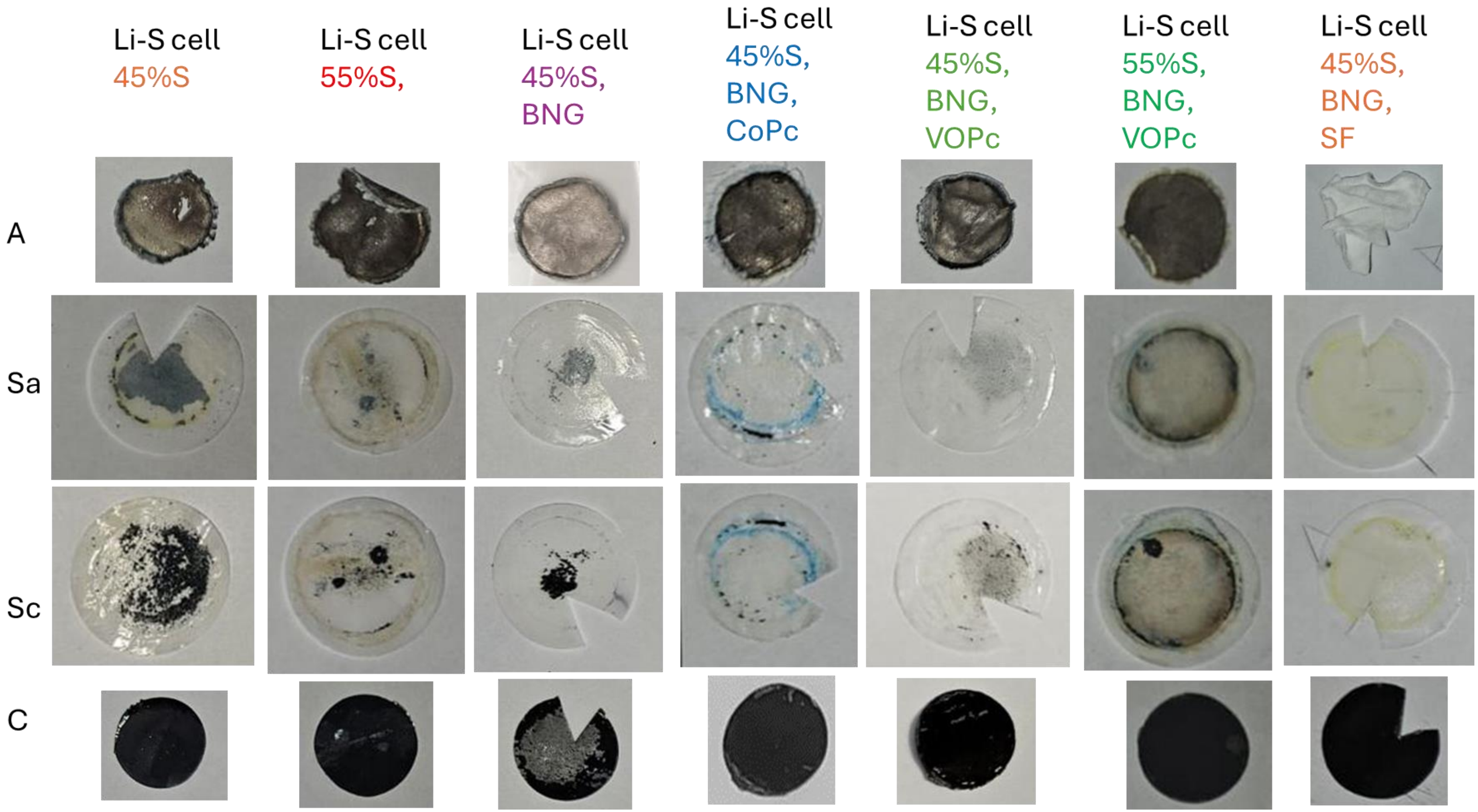

Figure S10. Postmortem photos of cell components after the full cycling schedule: A = anode, Sa = separator surface facing the anode, Sc = separator surface facing the cathode, C = cathode.

2.6 Results of multiscale modeling and simulations

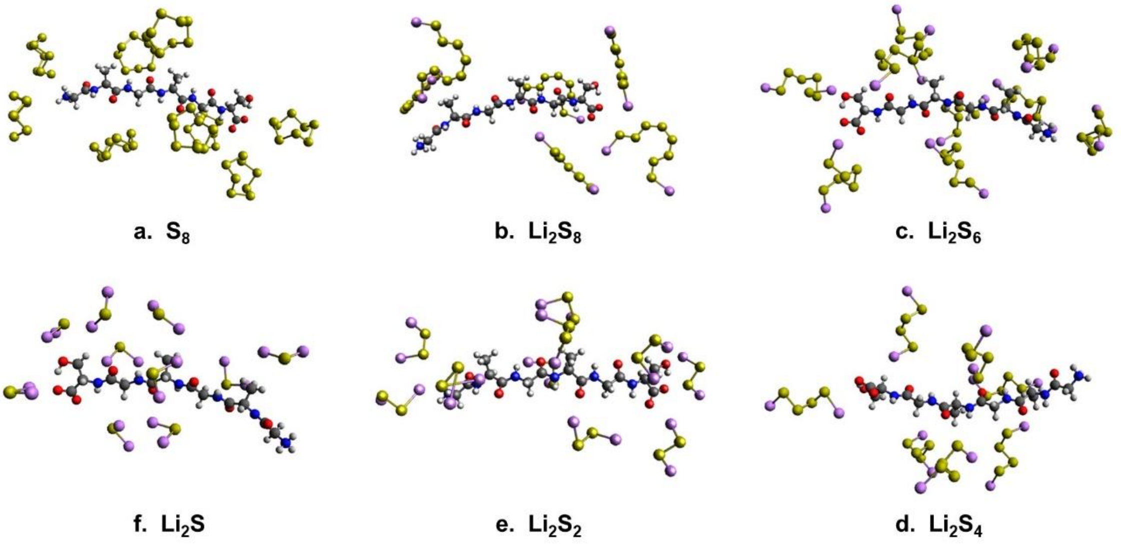

Figure S11. Structures of silk fibroin fragment and coordinated sulfur or lithium sulfide molecules after geometrical and energy minimization simulations using the Blends module of Materials Studio 6.1 (Accelrys, US).

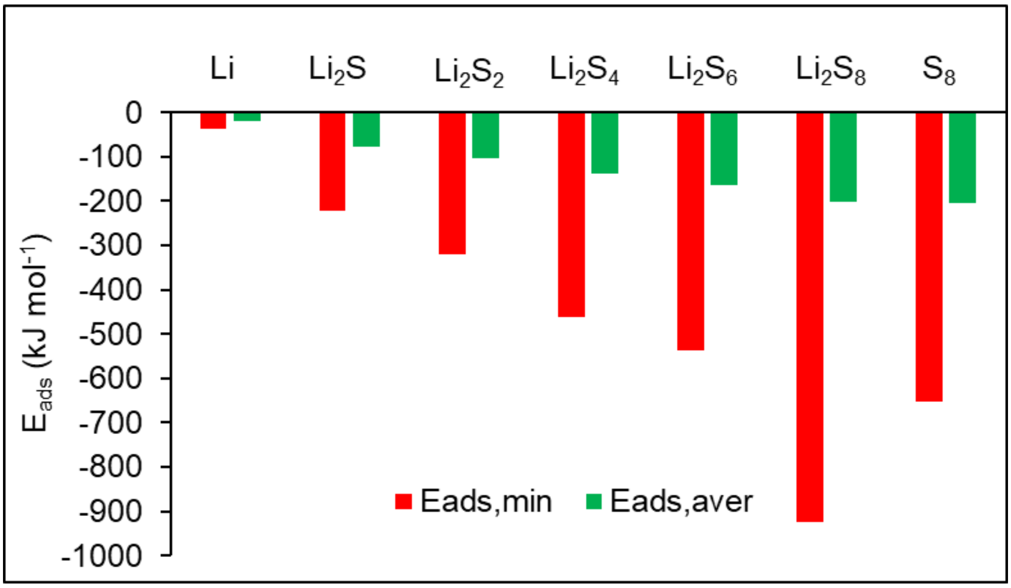

Figure S12. Adsorption energies (minimum and average) of coordinated sulfur and lithium sulfides adsorbed by the silk fibroin fragment (see Figure S11), as predicted by the MD simulations conducted using the Blends module of Materials Studio 6.1 (Accelrys, US). The average values, Eads,aver, were inputted in the continuum model based simulations in this study.

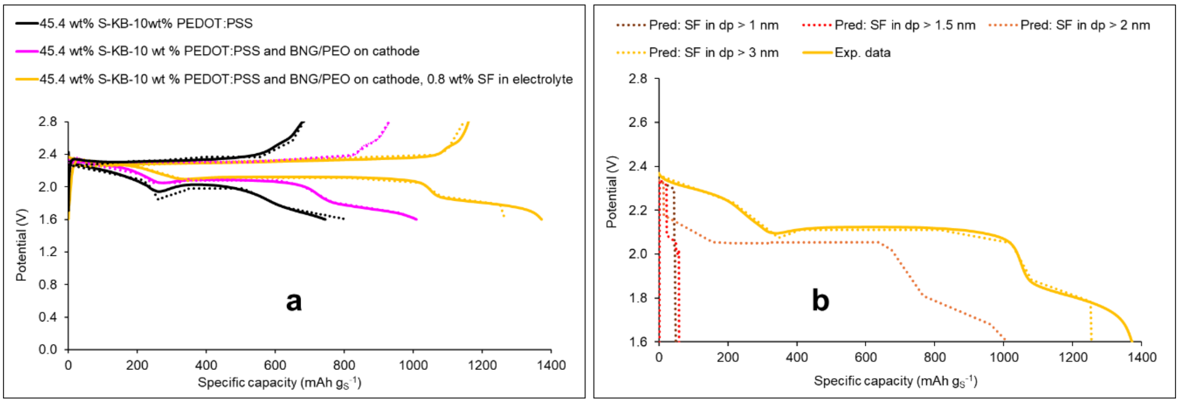

Figure S13. Predictions using the multipore continuum model (broken lines) against experimental data (solid lines) of the first GDC cycle at 0.05 C for Li-S cells with 45.4 wt% S-KB-10 wt% PEDOT:PSS cathode and BNG interlayer on the cathode surface; also with 0.8 wt% SF in the electrolyte for Li-S cell with GDC curve in deep yellow in **a.** and all curves in **b. b.** Predictions of first discharge at 0.05 C (against experimental data) for Li-S cell with 45.4 wt% S-KB-10 wt% PEDOT:PSS cathode and BNG interlayer on the cathode surface, and also 0.8 wt% SF in the electrolyte: predictions for different settings in the continuum model based simulations: SF in pores of dp > 1 nm or in dp > 1.5 nm or in dp > 2 nm or in dp > 3 nm.

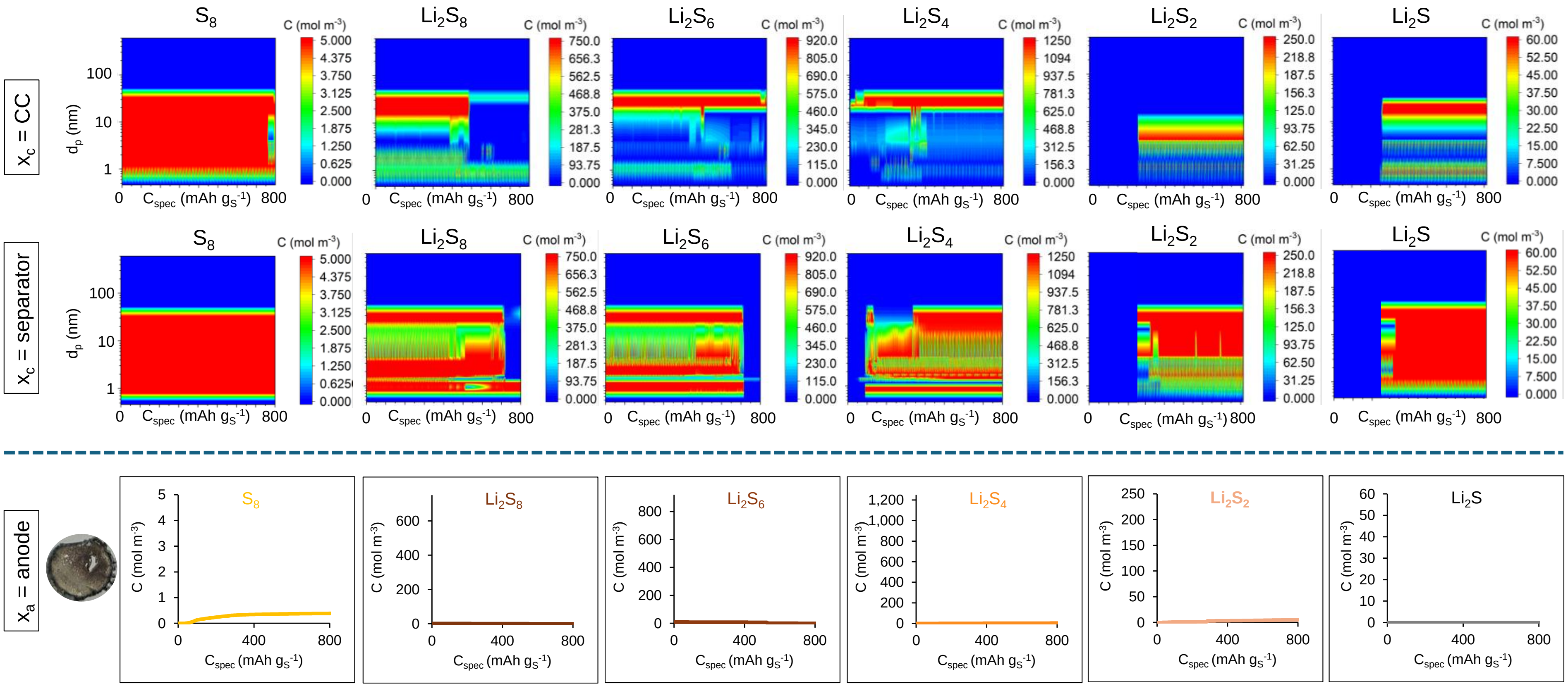

Figure S14. Predicted concentrations of the dissolved sulfur and sulfides in the liquid electrolyte as a function of specific capacity during the first discharge of the Li–S battery cell with cathode of 45.4 wt% S-KB-10 wt% PEDOT:PSS coating: contour plots in cathode as a function of pore size for two different locations (by the current collector and by the separator) and concentration profiles at anode together with a postmortem photo of the anode after the full cycling schedule. The maximum concentration limit in each plot is set to the saturation concentration of that species in the electrolyte solution.
